# Microstructural Evolution and Mechanical Properties of Investment-Cast Haynes 282 Nickel-Based Superalloy After Heat Treatment and High-Temperature Thermomechanical Processing

**DOI:** 10.3390/ma19153282

**Published:** 2026-08-03

**Authors:** Andrzej Nowotnik, Elzbieta Wichowska, Grazyna Mrowka-Nowotnik

**Affiliations:** 1 Department of Material Science, Rzeszow University of Technology, Al. Powstancow Warszawy 12, 35-959 Rzeszow, Poland; mrowka@prz.edu.pl; 2 Doctoral School, Rzeszów University of Technology, 35-959 Rzeszów, Poland; elzbieta.wichowskaa@gmail.com

**Keywords:** Haynes 282, nickel superalloys, precision casting, thermomechanical processing, hot pressing, dynamic recrystallization, hardness, microstructure

## Abstract

This study analyzed the effect of a processing sequence comprising precision casting, heat treatment, and high-temperature plastic deformation on the microstructure and mechanical properties of the Haynes 282 nickel superalloy. The starting material was prepared in an industrial VIM IC induction furnace under vacuum conditions; the quality of the resulting castings, phase composition, thermal effects, and the alloy’s behavior during uniaxial compression were then evaluated. Castings in the form of rods with diameters of 10, 12, and 16 mm were produced at a molten alloy temperature of 1550 °C and a ceramic mold temperature of 1250 °C. The lowest porosity values, ranging from approximately 0.036–0.16%, were obtained for the vacuum furnace cooling variant, which was selected for further testing. DTA analysis revealed characteristic thermal effects in the range of 935.9–1379.8 °C, which enabled the selection of supersaturation parameters and a safe range for deformation tests. After supersaturation and aging, the samples were compressed at temperatures of 700–1200 °C at strain rates of 0.001 s^−1^ and 0.008 s^−1^. An increase in temperature caused a systematic decrease in maximum stress and yield stress, with the highest plastic resistance observed at temperatures of 700–800 °C. In this range, the microstructure exhibited characteristics of strong strain hardening, high dislocation density, and strain localization. At temperatures of 850–1000 °C, a transition to conditions of intense dynamic recovery and dynamic recrystallization was observed, whereas above 1050 °C, grain growth following recrystallization dominated. The most favorable compromise between reducing deformation resistance, minimizing the risk of cracking, and maintaining a finer microstructure was achieved in the 900–1000 °C range. The results indicate that the combination of precision casting and controlled thermomechanical working can serve as the basis for further optimization of the manufacturing technology for Haynes 282 superalloy semi-finished products.

## 1. Introduction

Nickel superalloys strengthened by γ′ phase precipitation are among the primary materials used in components operating under high-temperature conditions, complex mechanical loads, and intense exposure to an oxidizing environment [1,2,3,4,5,6,7,8]. The requirements for hot-section components in aircraft engines and industrial turbines include high heat resistance, creep resistance, microstructural stability, and the ability to undergo complex manufacturing processes such as casting, forging, rolling, welding, and heat treatment.

Haynes 282 is a nickel superalloy developed as a material offering a favorable compromise between high-temperature strength and processability [8,9,10,11]. This alloy is precipitation-hardened by the γ′ phase and is intended for high-temperature structural applications, particularly in aircraft engines, industrial gas turbines, and power generation systems. The literature emphasizes that its appeal stems from a combination of creep resistance, thermal stability, and good weldability, resulting, among other factors, from its moderate γ′ phase content and the relatively slow kinetics of its precipitation [8,9,10,11,12].

Although Haynes 282 is primarily associated with wrought materials—that is, semi-finished products intended for plastic deformation—in recent years, attempts have also been made to use this alloy in casting technologies [13,14,15,16,17,18,19,20,21,22]. This trend stems from the need to manufacture components with more complex geometries, the production of which using only conventional plastic deformation methods would be difficult or technologically unfeasible. Research conducted, among other things, as part of the A-USC advanced power unit programs included an evaluation of Haynes 282’s castability, its fluidity, the quality of the resulting castings, microsegregation, and the ability to achieve a microstructure acceptable for further heat treatment and operation under high-temperature conditions [13].

Also of significant importance are the studies by Matysiak and colleagues, in which Haynes 282 alloy was cast using the VIM IC method into thin-walled ceramic molds. The authors used a mold temperature of 1250 °C and a pouring temperature of 1550 °C—parameters similar to those used in this study. It was demonstrated that with proper control of the molten metal temperature, mold temperature, and solidification conditions, it is possible to produce thin-walled castings from the Haynes 282 alloy without critical casting defects such as short shots, hot cracks, significant porosity, or non-metallic inclusions [23]. This confirms that, despite the alloy’s original intended use in plastic deformation, its application in precision casting is technologically feasible.

At the same time, studies on large Haynes 282 castings indicate that the casting process itself does not eliminate problems typical of the solidification microstructure. Yang and colleagues demonstrated that the microstructure of large Haynes 282 castings may include a γ matrix, a γ′ phase, MX-type carbides, M_23_C_6_, and molybdenum-rich phases, with heat treatment leading to significant changes in the stability and distribution of these components [24]. This means that good castability of the alloy does not necessarily result in a microstructure that is optimal in terms of mechanical properties. In the as-cast condition, the material may still exhibit dendritic segregation, the presence of primary and secondary carbides, local variations in chemical composition, and a non-uniform distribution of secondary phases.

For this reason, studies comparing the properties of Haynes 282 in the as-cast and cold-worked states are particularly important. Kim and colleagues demonstrated that as-cast Haynes 282 may exhibit lower strength and significantly lower ductility compared to the cold-worked material, which was attributed to the presence of microsegregation, larger precipitates, microstructural inhomogeneities, and defects formed during solidification [19]. At the same time, creep test results indicate that, following appropriately selected heat treatment, the high-temperature properties of the cast material can approach those of wrought material. This indicates that not only the casting process itself is of key importance, but also the subsequent shaping of the microstructure through heat treatment and possible thermomechanical processing.

In this context, this paper does not treat Haynes 282 as a classic casting alloy intended for direct use in its as-cast state. Instead, it is assumed that precision casting can be used as a stage in the production of a semi-finished product with controlled geometry, which is then subjected to heat treatment and controlled hot deformation. This assumption stems both from the alloy’s properties and from the ability to minimize casting defects under VIM IC conditions. The addition of boron and silicon can promote crystallization, improve fluidity, and stabilize grain boundaries, while aluminum and titanium enable subsequent control of γ′-phase precipitation hardening.

In this study, trial castings of model turbine blades were first produced to verify the ability of the Haynes 282 alloy to fill thin-walled ceramic molds. These tests allowed for an assessment of the limitations related to the alloy’s fluidity and the potential for premature solidification in areas with thin walls. Subsequently, after modifying the process parameters, castings of bars with diameters of 10, 12, and 16 mm were produced, which served as the starting material for further heat treatment and high-temperature compression tests. This approach enabled a transition from evaluating the alloy’s castability alone to analyzing its suitability as a cast semi-finished product intended for further microstructure shaping using thermomechanical processing methods.

Thermal-plastic processing tests were therefore conducted to determine whether the material obtained by precision casting could serve as a technologically useful semi-finished product for further microstructural modification. This experimental setup also allows for the simultaneous assessment of which microstructural features, after casting, are constrained by heat treatment and deformation, and which continue to govern the mechanisms of flow, strain localization, cracking, and recrystallization during high-temperature deformation [25,26].

From the perspective of hot plastic deformation, it is crucial to identify the temperature range in which it is possible to reduce deformation resistance while simultaneously avoiding strain localization, cracking, and excessive grain growth. In nickel superalloys, the mechanisms of strain hardening, the interaction of dislocations with γ′ precipitates and carbides, dynamic recovery, and dynamic recrystallization compete with one another. Proper control of temperature and strain rate allows the microstructure to be transformed from a heavily deformed state, through a partially or fully recrystallized state, to a coarse-grained structure at excessively high deformation temperatures. In this respect, the analysis of hot deformation behavior is commonly supported by constitutive descriptions based on the Zener–Hollomon parameter, Arrhenius-type equations, strain hardening rate analysis and processing maps [27,28,29,30,31,32,33,34]. These approaches make it possible to relate flow stress, strain rate and deformation temperature to the onset of dynamic recovery, dynamic recrystallization and flow instability. In nickel-based superalloys, such analyses have been widely applied to identify safe hot-working windows, determine apparent activation energies and correlate the shape of flow curves with microstructural mechanisms such as dislocation rearrangement, grain-boundary bulging, subgrain formation and recrystallized grain growth [35,36,37,38,39,40,41,42,43,44,45]. Recent studies on Haynes 282 produced by additive or powder-based routes further confirm that the final mechanical response of this alloy is strongly controlled by the initial processing route, heat treatment, γ′ precipitation state, carbide distribution, porosity and the degree of microstructural homogeneity [46,47,48,49]. Therefore, the analysis of investment-cast Haynes 282 as a semi-finished product for subsequent thermomechanical processing should be considered in the broader context of microstructure–processing–property relationships already established for nickel-based superalloys.

### Current State of Research and Novelty of This Study

Previous research has confirmed that the individual stages of the analyzed production process have already been examined separately. Matysiak et al. demonstrated the possibility of producing thin-walled precision castings from Haynes 282 alloy using the vacuum induction melting method and casting into ceramic molds [13]. In turn, Thangirala and Myers analyzed the production of large-sized sand castings of this alloy intended for use in advanced power systems [14]. Yang’s research focused on the microstructural evolution of large Haynes 282 castings during prolonged exposure to elevated temperatures and on optimizing heat treatment to minimize cracks initiated at grain boundaries and in carbide regions [15,16]. The literature also describes the influence of shrinkage porosity and local segregation on γ′ phase precipitation [17], as well as the effect of a heterogeneous casting microstructure on tensile and creep properties [18]. Kim et al. compared the properties of Haynes 282 alloy in the as-cast and cold-worked states, demonstrating lower strength and ductility in the cast material, while noting relatively minor differences in its creep resistance following appropriately selected heat treatment [19]. Dynamic and post-dynamic recrystallization of Haynes 282 alloy during deformation below the solvus temperature of secondary carbides was also studied, indicating a significant influence of deformation conditions and secondary particles on the course of microstructure recovery [50]. The results of these studies show that casting, heat treatment, or hot deformation of Haynes 282 alone do not constitute new research topics in and of themselves.

The novelty of this work therefore does not lie in the application of individual, previously known technological operations, but rather in their sequential combination and a comprehensive assessment of the interrelationships between the microstructure formed during precision casting, its modification during heat treatment, and the mechanisms of deformation and microstructural regeneration activated during subsequent hot pressing. In contrast to studies that analyzed Haynes 282 material in a plastically deformed state or limited the evaluation of castings to their quality, phase stability, and properties after heat treatment, in this work the cast microstructure was treated as a controlled initial state for further thermomechanical shaping. A key aspect of the research is the correlation of changes in yield stress, hardness, and mechanical properties with the evolution of grains, γ′ phase precipitates, and carbides, as well as with the transition from strain hardening and dynamic recovery to partial or complete dynamic recrystallization. This approach makes it possible to assess the extent to which controlled thermomechanical processing can mitigate the adverse effects of microsegregation and inhomogeneities characteristic of the as-cast condition and transform a precision casting into a technologically useful semi-finished product intended for further forming.

The objective of this study was to evaluate the potential of combining precision casting, heat treatment, and high-temperature plastic deformation to control the microstructure and mechanical properties of the Haynes 282 superalloy. Particular attention was paid to the influence of temperature and strain rate on the flow curves, hardness, mechanical properties determined after prior compression, and the microstructure observed using LM, SEM, and TEM methods.

## 2. Materials and Methods

**Figure 1:**
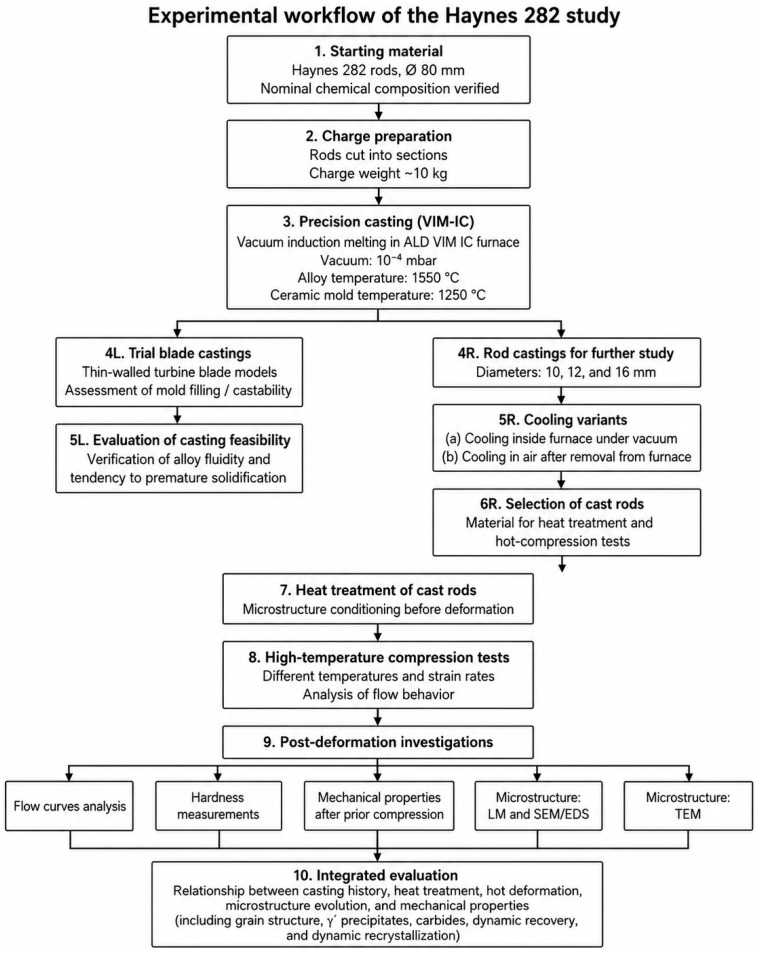
Schematic representation of the experimental workflow applied to the Haynes 282 superalloy, comprising preparation of the starting material, VIM-IC investment casting of trial blades and rods, evaluation of casting feasibility and cooling conditions, selection and heat treatment of cast rods, high-temperature compression testing, flow-curve analysis, hardness and tensile testing, and LM, SEM/EDS, and TEM investigations.

The complete experimental workflow applied in this study is presented in Figure 1. The research procedure included preparation of the Haynes 282 charge, vacuum investment casting of trial turbine blades and rods, evaluation of the casting and cooling variants, heat treatment of the selected cast material, high-temperature compression testing, and subsequent mechanical and microstructural characterization. The individual stages of the experimental procedure are described in detail in Section 2.1, Section 2.2 and Section 2.3.

### 2.1. Test Material and Precision Casting

Haynes 282 nickel superalloy, supplied in the form of rods with a diameter of 80 mm, was used for the tests. Charges for the individual casting processes were prepared by cutting the rods into sections to obtain a charge weight of approximately 10 kg. The nominal chemical composition of the alloy is shown in Table 1.

**Figure 2:**
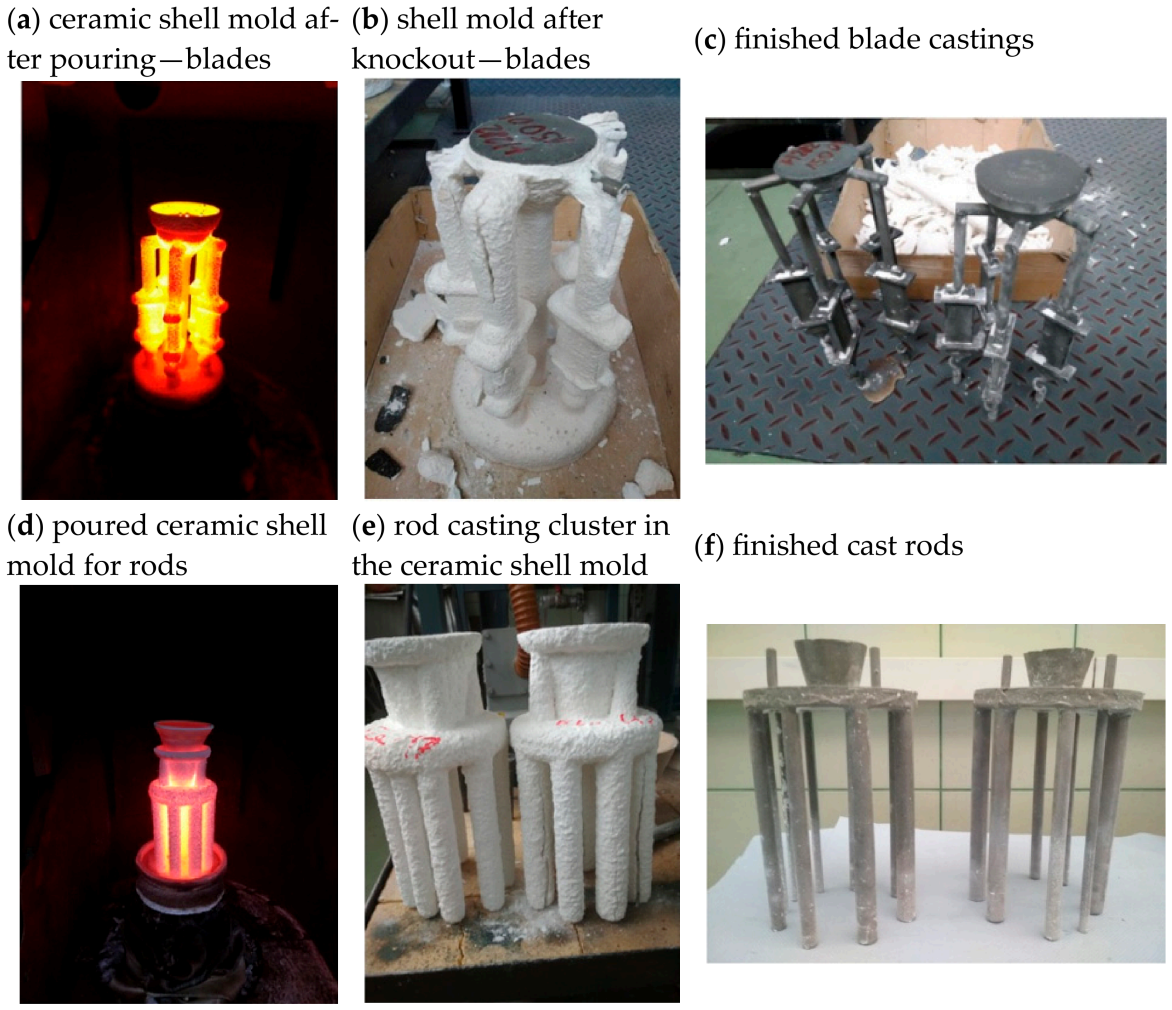
Photographic documentation of the stages of preparation of the research material: (**a**–**c**) model turbine blade castings; (**d**–**f**) Haynes 282 rod castings intended for further mechanical and microstructural investigations.

The casting process was carried out in an industrial vacuum induction melting (VIM) furnace, model IC, manufactured by ALD Vacuum Technologies GmbH (Hanau, Germany). Melting was performed under a vacuum of 10^−4^ mbar in a 150-mm-diameter ceramic crucible, and the molds were filled using an automatic pouring system. In the preliminary stage, test castings of turbine blades were produced to evaluate the alloy’s ability to fill thin-walled ceramic molds. Subsequently, castings of rods with diameters of 10, 12, and 16 mm were produced for further mechanical and microstructural testing. The actual castings were produced at a liquid alloy temperature of 1550 °C and a ceramic mold temperature of 1250 °C. The relatively high mold temperature was selected to ensure sufficient melt fluidity and complete filling of the casting geometry, including thin blade sections and small-diameter rods, while limiting premature solidification during pouring. It also reduced the temperature gradient and thermal shock between the molten alloy and the ceramic mold. Two cooling methods were compared: cooling the filled mold inside the furnace under vacuum conditions and cooling after removal from the furnace at ambient temperature. Representative stages of ceramic mold preparation, pouring, and the resulting castings are shown in Figure 2.

### 2.2. Casting Evaluation, Thermal Analysis, and Heat Treatment

The quality of the castings was evaluated on metallographic sections prepared in accordance with the grinding and polishing procedure. A quantitative analysis of porosity was performed using image analysis in ImageJ software (version 1.54f, National Institutes of Health, Bethesda, MD, USA) on seven randomly selected observation fields for each sample. The phase composition was analyzed by X-ray diffraction in Bragg–Brentano geometry, using CuKα radiation, in the range of 2θ = 20–80°, with a step size of 0.02°. The XRD analysis was qualitative and semi-quantitative, and its purpose was to confirm the presence of the gamma matrix, the gamma prime phase, and potential secondary phases.

Characteristic transformation temperatures were determined using the DTA/DSC method with a NETZSCH STA 449 F1 Jupiter analyzer (Selb, Germany). Measurements were conducted in an argon atmosphere in the range of 25–1500 °C, at a heating rate of 10 °C/min. The cooling stage was not performed at the same programmed rate and proceeded naturally in the furnace. Therefore, the characteristic transformation temperatures reported in this study were determined from the heating curve only. Heat treatment parameters were selected based on the results of the thermal analysis. Rods with a diameter of 12 mm were subjected to supersaturation at 990 °C for 1 h in a vacuum of 10^−5^ mbar, after which they were cooled with argon gas at a pressure of 8 bar. The temperature of 990 °C was selected based on the DTA/DSC results, which indicated pronounced thermal effects associated with changes in the γ′ precipitates and carbides in the range of approximately 935–1000 °C. This treatment was not intended to produce complete γ′ dissolution but rather to modify the initial precipitation state while preserving the characteristic cast microstructure before hot-compression testing. This was followed by aging at 760 °C for 5 h and cooling with argon at a pressure of 2 bar. The material prepared in this manner was used for high-temperature compression tests.

### 2.3. Compression, Tensile, and Microstructural Tests

Uniaxial compression tests were performed using a Gleeble 3800 thermal simulator in an argon atmosphere (Dynamic Systems Inc., Poestenkill, NY, USA). The specimens were heated to the specified deformation temperature, held for 5 min to minimize the temperature gradient, and then deformed in the range of 700–1200 °C at two deformation rates: 0.001 s^−1^ and 0.008 s^−1^. During the tests, true stress–true strain curves were recorded. The maximum flow stress and the corresponding strain were determined directly from each curve. The initial increasing section of the flow curves, the occurrence of a stress maximum, subsequent flow softening, and the tendency toward stress stabilization at higher strains were analyzed qualitatively. These features were correlated with the microstructural observations to discuss the relative contributions of strain hardening, dynamic recovery, and dynamic recrystallization. No separate quantitative work-hardening-rate analysis or determination of the critical DRX strain using the Poliak–Jonas method was performed.

Flat microspecimens were cut from the central part of the specimens after compression for static tensile testing at room temperature. The tests were conducted at a strain rate of 10^−3^ s^−1^, determining the yield strength R_p0.2_, tensile strength R_m_, and relative elongation A. Hardness measurements were performed using the Vickers method at a load of HV10 and a loading time of 10 s. Five measurements were performed for each variant, and the results were presented as the mean value ± standard deviation.

Microstructural observations were conducted using light microscopy, scanning electron microscopy, and transmission electron microscopy. Metallographic specimens were etched with AG21 reagent. SEM imaging was performed at a voltage of 10–20 kV in SE and BSE modes. TEM analyses were conducted on thin films prepared by the FIB method from the central regions of the samples after compression. Cautious wording was used in the interpretation of secondary precipitates, as unambiguous identification of carbide phases requires full correlation of TEM images, SAED, and EDS analyses.

### 2.4. Statistical Analysis

Quantitative results are presented as the mean value ± standard deviation. Porosity was determined from seven randomly selected observation fields for each specimen. Five Vickers hardness measurements were performed for each material condition, while three tensile microspecimens were tested for each investigated deformation condition. Error bars in the corresponding figures represent one standard deviation. Due to the limited amount of available cast material, the Gleeble hot-compression tests were performed using a single specimen for each combination of deformation temperature and strain rate. However, the repeatability of the procedure was additionally verified for two extreme deformation conditions. The repeated tests produced closely comparable flow curves and characteristic stress values, confirming satisfactory repeatability of the hot-compression experiments. Consequently, standard deviations and error bars could not be determined for all Gleeble test conditions. XRD, DTA/DSC, LM, SEM, and TEM analyses were used primarily for qualitative phase and microstructural characterization; therefore, statistical error bars were not applicable to these results.

## 3. Results

### 3.1. Casting Quality and Phase Composition

The castings of model blades and rods obtained after optimizing the process parameters were characterized by accurate reproduction of the ceramic mold geometry and the absence of visible macroscopic surface defects. However, macroscopic evaluation alone was not sufficient to qualify the material for further testing, since in nickel superalloys even a small amount of microporosity can lead to local stress concentrations during high-temperature deformation. Therefore, the porosity fraction was determined for rods cast under two different cooling conditions (Table 2).

The vacuum furnace cooling variant showed low porosity levels for all analyzed rod diameters. For rods with diameters of 10, 12, and 16 mm, the porosity values were 0.036%, 0.160%, and 0.088%, respectively. For air cooling, the values were 0.078%, 0.062%, and 0.100%, respectively. The results indicate that there was no clear correlation between bar diameter and porosity. However, the vacuum furnace cooling method was selected for further research because it ensured stable crystallization conditions and minimized the risk of atmospheric effects on the casting.

**Figure 3:**
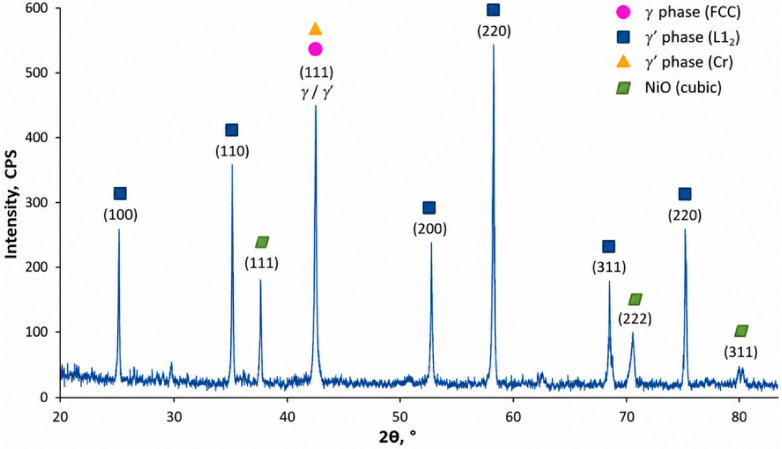
XRD diffractogram of the Haynes 282 alloy in the as-cast state. The pattern confirms the presence of the g matrix and g’ Ni_3_(Al,Ti) phase; low-intensity reflections may be associated with secondary phases and/or surface oxidation products. Phase identification was performed using ICDD PDF cards 00-004-0850 for the γ-Ni matrix, 00-009-0097 for the γ′-Ni_3_(Al,Ti) phase, and 00-047-1049 for NiO.

XRD analysis of the alloy in the as-cast condition confirmed the presence of the γ matrix and reflections attributable to the γ′-Ni_3_(Al,Ti) phase. However, because the diffraction peaks of the γ and γ′ phases are closely located and partially overlap, their relative intensities cannot be used as a reliable quantitative measure of the γ′ phase fraction. Moreover, Figure 3 presents only the as-cast condition and does not provide a direct comparison of the material before and after the heat treatment at 990 °C. Therefore, the interpretation of partial changes in the γ′ precipitation state after this treatment was based primarily on the DTA/DSC results rather than on the XRD pattern alone (Figure 3).

### 3.2. Thermal Analysis and Preparation of the Material for Deformation

**Figure 4:**
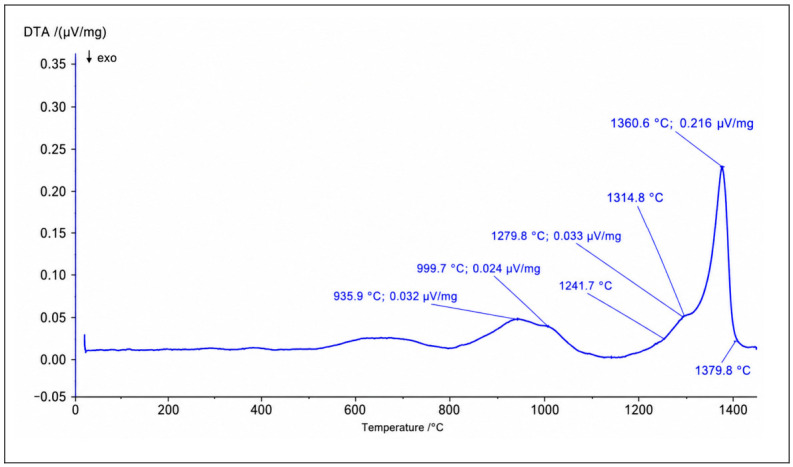
DTA/DSC curve of the as-cast Haynes 282 alloy showing characteristic thermal effects recorded during heating. The marked effects were used to select the solution treatment temperature and to define the safe temperature range for subsequent high-temperature compression tests.

Thermal effects were observed on the DTA/DSC curve (Figure 4) at temperatures of 935.9 °C, 999.7 °C, 1241.7 °C, 1279.8 °C, 1314.8 °C, 1360.6 °C, and 1379.8 °C. According to the signal convention used in Figure 4, the exothermic direction is downward; therefore, the effects recorded at 935.9 °C and 999.7 °C are endothermic. These effects were associated with progressive dissolution or destabilization of secondary precipitates during heating, primarily involving the γ′ phase. Because the available data do not allow an unambiguous assignment of either effect to a specific carbide transformation, such an interpretation was not made. The 1240–1315 °C range corresponded to a further intensification of the dissolution processes of secondary constituents and the material’s approach to the region of high diffusion activity. The strongest endothermic effect observed near 1360.6 °C indicates the onset of incipient melting of microstructural constituents and the approach to the liquidus range. The DTA/DSC results served as the basis for selecting a pre-deformation heat-treatment temperature and supersaturation temperature of 990 °C. After supersaturation, the material’s hardness decreased from an average of 330 HV10 in the as-cast condition to 259 HV10. This decrease in hardness confirms the partial dissolution of secondary precipitates and the homogenization of the γ- matrix. This behavior is consistent with the expected reduction in the γ′ volume fraction at 990 °C, which is close to but still below the γ′ solvus of Haynes 282. Therefore, partial dissolution of γ′ is expected, whereas complete dissolution should not occur under these conditions. However, because the γ′ volume fraction was not measured quantitatively in the present study, this interpretation remains qualitative, with the treatment simultaneously preparing the material for further compression testing under comparable initial conditions.

The significance of the DTA/DSC analysis therefore lay not only in determining the alloy’s characteristic temperatures, but above all in establishing a safe and technologically justified range of deformation temperatures. The compression test range of 700–1200 °C was selected to cover three different microstructural and mechanical states of the material: the low-temperature range, in which a strong interaction between γ′-precipitates and carbides with dislocations, as well as high deformation resistance, was expected; the intermediate range, in which dynamic recovery and dynamic recrystallization could be activated; and the high-temperature range, in which the risk of excessive grain growth had to be assessed while maintaining a safe distance from the remelting temperatures. This approach made it possible to directly link the results of the thermal analysis to the design of the Gleeble tests and to the subsequent interpretation of the flow curves and microstructural evolution.

### 3.3. Behavior of the Alloy During High-Temperature Compression

**Figure 5:**
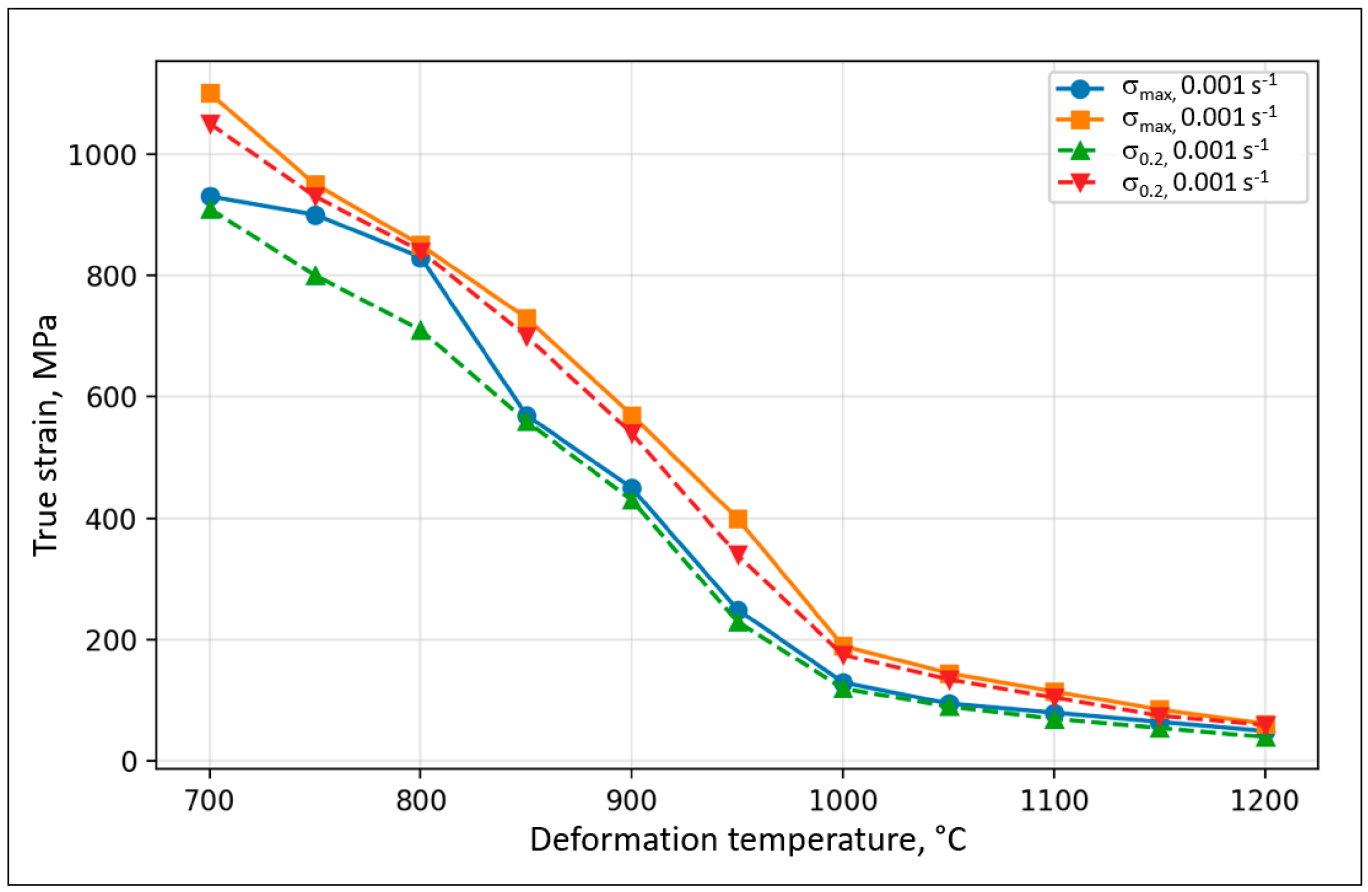
Dependence of maximum stress and yield stress on deformation temperature for two strain rates.

Based on the DTA/DSC results and the selected heat-treatment conditions, high-temperature compression tests were carried out using a Gleeble thermomechanical simulator. The aim of these tests was to determine the response of the investment-cast and heat-treated Haynes 282 alloy to hot deformation and to identify the temperature range in which the deformation resistance decreases without excessive microstructural degradation. The analysis focused on the maximum flow stress, σ_max_, and the yield stress, σ_0.2_, determined from the compression tests and summarized as a function of deformation temperature and strain rate (Table 3, Figure 5). An increase in deformation temperature led to a marked and systematic decrease in maximum stress and yield stress. For a strain rate of 0.001 s^−1^, the value of σ_max_ decreased from 930 MPa at 700 °C to 50 MPa at 1200 °C. For a strain rate of 0.008 s^−1^, these values were 1100 MPa and 62 MPa, respectively. Across the entire temperature range, a higher strain rate caused an increase in plastic resistance, which is consistent with the limitation of the time available for diffusive mechanisms of strain energy relaxation.

In the 700–850 °C range, the stress values indicate a high resistance of the alloy to plastic deformation. This behavior indicates intense strain hardening, strong interaction of dislocations with γ′ precipitates and carbides, and the limited contribution of dynamic recovery. Above 900 °C, the stress level decreased significantly, indicating an increasing contribution of thermally activated restoration processes, including dynamic recovery and the onset of dynamic recrystallization. The highest deformation temperatures, 1150–1200 °C, corresponded to conditions in which microstructural restoration processes were highly active; however, at these temperatures grain growth may also begin to dominate.

### 3.4. Activation Energy of the Deformation Process

The activation energy of the high-temperature deformation process was determined to quantitatively assess the effect of temperature and strain rate on the plastic resistance of the Haynes 282 superalloy. This parameter allows for an evaluation of the energy required to activate the mechanisms responsible for the plastic flow of the material under elevated-temperature conditions. In the case of precipitation-hardened nickel superalloys, the activation energy value should not be interpreted as a parameter associated exclusively with a single mechanism, such as volume diffusion, but rather as apparent activation energy, reflecting the combined contribution of several processes occurring simultaneously during deformation. These include dislocation motion and climb, the interaction of dislocations with γ′ phase precipitates and carbides, dynamic recovery, dynamic recrystallization, and, at the highest temperatures, grain boundary migration and grain growth.

The activation energy was determined based on the relationship between yield stress, deformation temperature, and deformation rate, in accordance with the approach used in the analysis of high-temperature deformation of metals and alloys. This method is based on an Arrhenius-type constitutive equation, in which the strain rate is a function of the yield stress and absolute temperature. In its general form, this relationship can be written as:

$$\overset{˙}{\varepsilon }=A[sinh(\alpha \sigma ){]}^{n}exp(-\frac{Q}{RT})$$

where: $\overset{˙}{\varepsilon }$ denotes the strain rate, σ is the assumed characteristic stress, T is the absolute temperature, R is the gas constant, and A, α, and *n* are material constants.

To determine the constitutive parameters, the Arrhenius relationship was analyzed using its linearized forms. The parameters n’ and β were determined from the slopes of the relationships between ln$\overset{˙}{\varepsilon }$ and lnσ and between ln$\overset{˙}{\varepsilon }$ and σ, respectively. The stress multiplier α was then calculated from the relationship:

α = β/n′

The stress exponent n was determined from the slope of the linear relationship between ln$\overset{˙}{\varepsilon }$ and ln[sinh(ασ)] at constant temperature. The apparent activation energy Q was calculated from the temperature dependence of ln[sinh(ασ)] at constant strain rate according to:

$$Q=Rn{\left(\frac{\partial ln[sinh(\alpha \sigma )]}{\partial (1/T)}\right)}_{\overset{˙}{\varepsilon }}$$

The Zener–Hollomon parameter, which combines the effects of strain rate and deformation temperature, was calculated as:

$$Z=\overset{˙}{\varepsilon }exp(\frac{Q}{RT})$$

The relationship between the Zener–Hollomon parameter and the characteristic stress was expressed as:

$$Z=A{\left[sinh(\alpha \sigma )\right]}^{n}$$

The material constant A was determined from the intercept of the linear regression of lnZ versus ln[sinh(ασ)]. The quality of the linear fittings was evaluated using the corresponding coefficients of determination, R^2^, which were also used to assess the reliability of the calculated constitutive parameters and apparent activation energy.

A similar approach was previously used in studies on high-temperature deformation of nickel alloys and steels, in which the activation energy of the deformation process was determined based on yield stresses from compression tests, and the influence of temperature and strain rate on the course of plastic flow was analyzed. In Nowotnik’s studies on the deformation of nickel alloys, it was shown that the stress–strain relationships obtained from high-temperature deformation tests can be used to determine the activation energy and to describe the influence of deformation parameters on the structural mechanisms occurring in the material [51].

**Figure 6:**
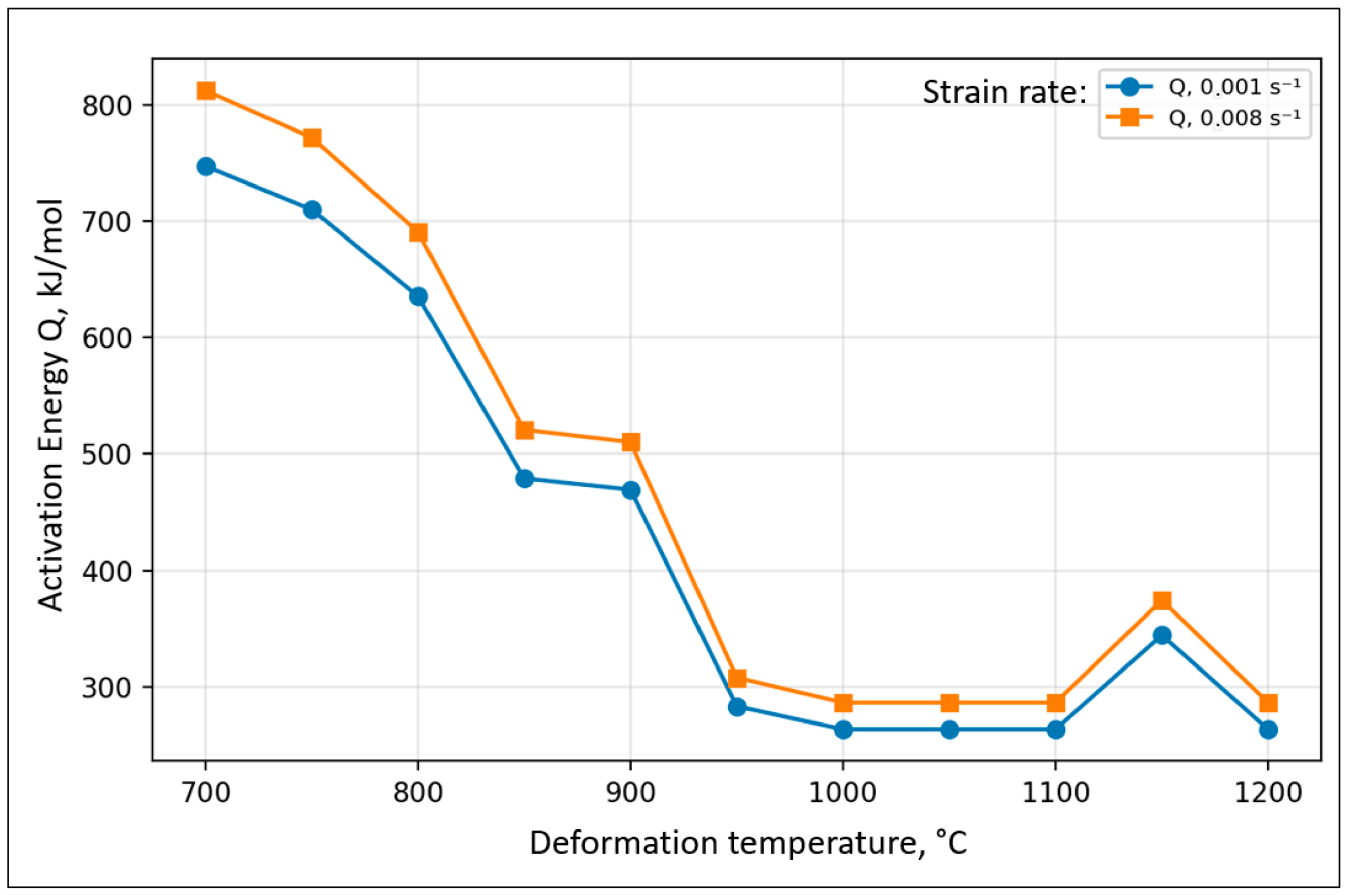
Dependence of the activation energy of plastic deformation on temperature for two strain rates.

The determined activation energy values showed a strong dependence on the deformation temperature (Figure 6). The highest Q values were obtained in the range of 700–800 °C and amounted to 747.09–635.35 kJ/mol for a strain rate of 0.001 s^−1^ and 812.06–690.61 kJ/mol for a strain rate of 0.008 s^−1^. Such high values indicate the material’s significant resistance to plastic deformation (Figure 6). This should be attributed primarily to the intense interaction of dislocations with γ′ phase precipitates and carbide particles, as well as to the limited activity of diffusion processes in this temperature range. Under such conditions, the material exhibits a tendency toward the accumulation of crystal lattice defects, an increase in dislocation density, and strong strain hardening, which is consistent with the high values of yield stress and hardness. The apparent activation-energy values obtained in this low-temperature range were considerably higher than those commonly reported for wrought Haynes 282 and related alloys. This difference may be attributed to several factors. First, the investigated material retained a heterogeneous cast microstructure containing dendritic and interdendritic regions, γ′ precipitates, and carbide particles, which increased the initial resistance to deformation. Second, the constitutive calculations were based on the yield stress rather than on the peak or steady-state stress. Because the yield stress represents the initial stage of plastic deformation, it is particularly sensitive to precipitation strengthening, dislocation–particle interactions, and local microstructural heterogeneity, which may increase the calculated apparent activation energy at 700–800 °C. Third, the calculations were performed over a relatively narrow temperature interval. Consequently, the slope used to determine Q was highly sensitive to small variations in stress, which may have magnified the resulting values. For comparison, apparent activation energies of approximately 537 kJ/mol have been reported for Haynes 282 [52], whereas a value of approximately 498 kJ/mol was reported for GH4282 [53], a wrought nickel-based superalloy regarded as an equivalent of Haynes 282. These literature values were obtained using broader hot-deformation datasets and characteristic stresses associated with later stages of flow. Directly comparable activation-energy data for investment-cast Haynes 282 remain limited. Therefore, the values obtained in the present study should be regarded as apparent activation energies specific to the investigated cast condition, the restricted low-temperature range, and the yield-stress-based calculation procedure rather than as intrinsic activation energies of a single deformation mechanism.

As the temperature increased to 900 °C, the activation energy decreased markedly, corresponding to a gradual transition from the dominance of strain hardening to conditions favorable for dynamic recovery and the initiation of dynamic recrystallization. The decrease in Q indicates that, as temperature increased, the efficiency of relaxation processes for energy stored during deformation also increased. Dislocations could more easily undergo rearrangement, annihilation, and substructure formation, and in regions of high strain energy concentration, new grain nucleation began.

Above 900 °C, the Q values stabilized at a lower level, which should be attributed to an increase in the mobility of dislocations and grain boundaries, as well as more efficient microstructure recovery processes. In this temperature range, dynamic recrystallization became the dominant mechanism shaping the alloy’s microstructure. The local increase in the Q value observed at a temperature of approximately 1150 °C may result from a change in the contribution of individual deformation mechanisms, in particular from the transition from intensive nucleation of new recrystallized grains to their subsequent growth. This interpretation is consistent with LM, SEM, and TEM observations, which revealed a transition from a highly deformed microstructure at lower temperatures, through a partially and then extensively recrystallized microstructure, to a coarse-grained structure at the highest deformation temperature.

### 3.5. Mechanical Properties After Deformation

The mechanical properties of Haynes 282 alloy after high-temperature deformation were evaluated to determine how the microstructure formed during a previous compression test affects the material’s subsequent behavior in a uniaxial tensile test at room temperature. This step was important because compression tests alone primarily describe the alloy’s yield strength under hot deformation conditions, whereas tensile tests performed after prior deformation allow for the evaluation of the mechanical properties retained after thermomechanical processing.

**Figure 7:**
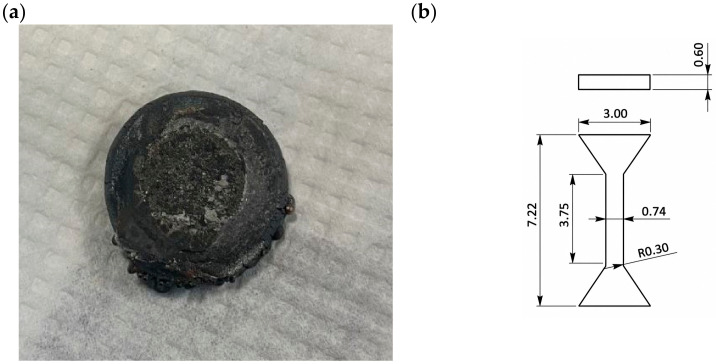
Specimens used to evaluate mechanical properties after high-temperature deformation: (**a**) a Haynes 282 superalloy specimen after a uniaxial compression test, (**b**) dimensions of a miniature specimen cut from the specimen after the compression process on a Gleeble thermosimulator, intended for determining mechanical properties in a uniaxial tensile test; dimensions are given in mm.

Miniature tensile specimens were prepared from the central part of the compression specimens, i.e., from the area corresponding to the most homogeneous state of plastic deformation. Figure 7 shows an example of a Haynes 282 superalloy specimen after a uniaxial compression test, as well as the geometry of a miniature specimen cut from this material for tensile testing. The use of this procedure allowed for the evaluation of mechanical properties directly in the region where deformation, dynamic recovery, and dynamic recrystallization occurred during the Gleeble test.

**Figure 8:**
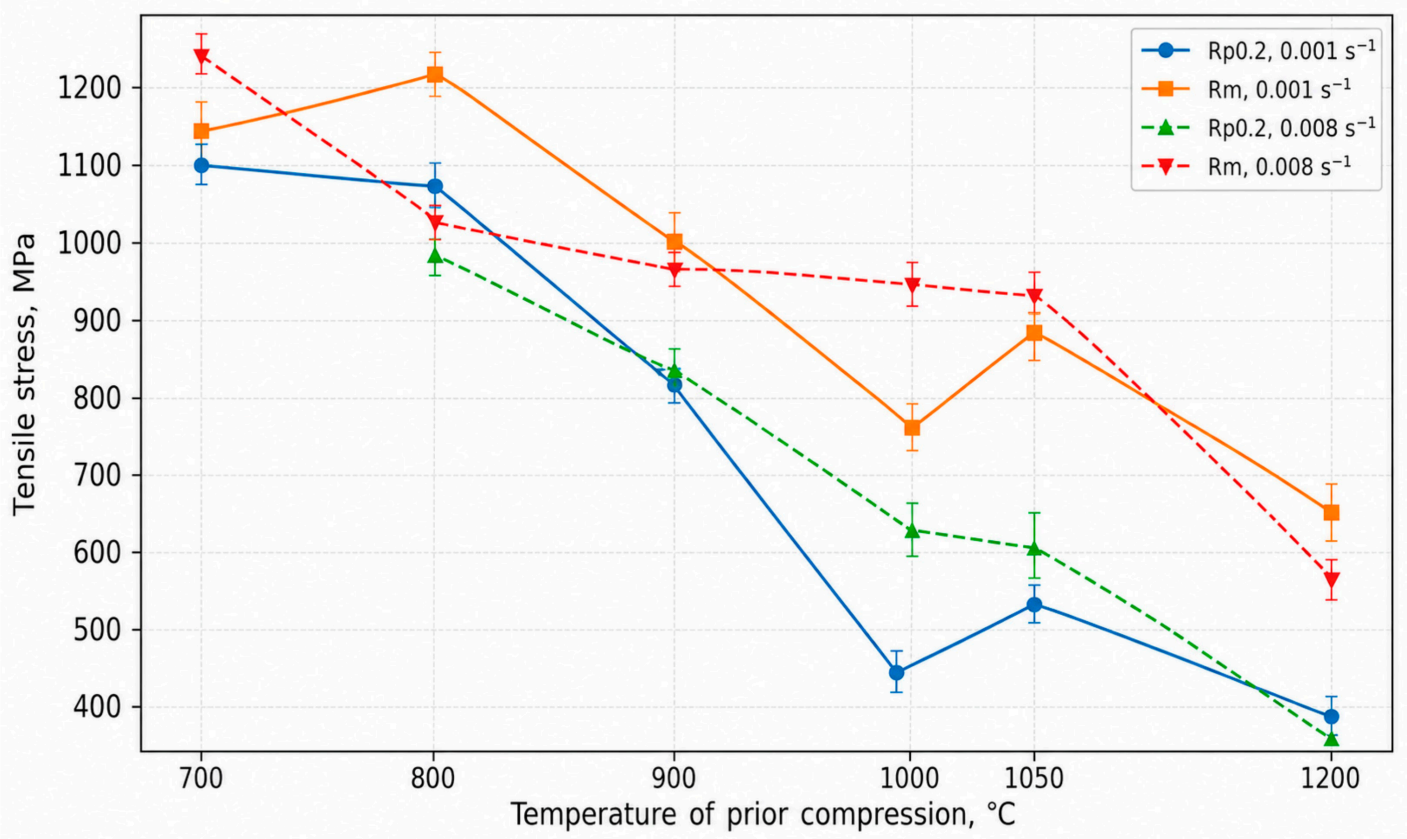
Changes in yield strength and tensile strength after prior compression at different temperatures.

**Figure 9:**
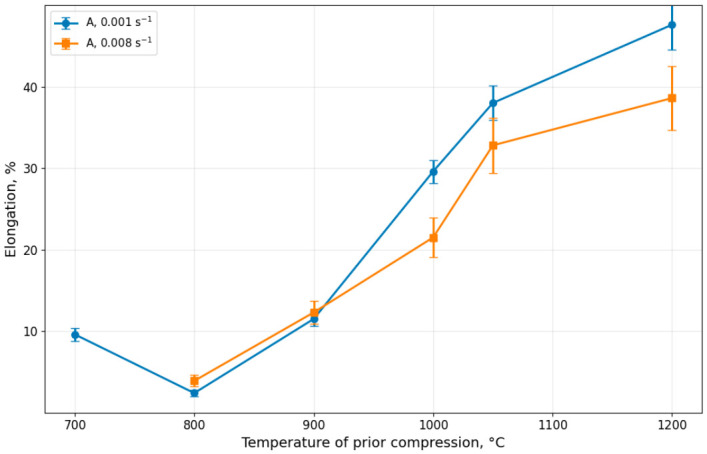
Changes in elongation after prior compression at different temperatures.

The results obtained should therefore be interpreted not as properties of the as-received material, but as the mechanical response of the alloy under specific heat-plastic working conditions. The analysis of the results focused on evaluating the balance between strain hardening and microstructure recovery processes. Deformation at a lower temperature should promote an increase in dislocation density, strong hardening, and a limited contribution of dynamic recovery, which leads to an increase in strength but simultaneously limits ductility. In contrast, deformation at a higher temperature activates dynamic recovery and dynamic recrystallization, which results in a decrease in strength but an improvement in the material’s ability to undergo further deformation. For this reason, the tensile test results were treated as an additional indicator of the effectiveness of the microstructural recovery processes occurring during prior high-temperature compression. A comparison of the alloy’s mechanical properties in the as-cast condition and after prior deformation under various temperature and strain rate conditions is presented in Table 4, while changes in yield strength, tensile strength, and elongation are shown in Figure 8 and Figure 9.

Specimens deformed at lower temperatures exhibited high strength with limited ductility. At a strain rate of 0.001 s^−1^, the highest R_m_ value, 1225 MPa, was obtained after prior compression at 800 °C, accompanied by a very low elongation of 2.4%. This result indicates strong strain hardening and limited capacity for further deformation. At 900 °C, the strength remained high, but the elongation increased to 11.5%, suggesting the onset of more effective microstructure recovery processes.

In the 1000–1200 °C range, a marked increase in ductility was observed, accompanied by a decrease in strength. At a strain rate of 0.001 s^−1^, elongation increased to 47.6% after compression at 1200 °C, but R_m_ decreased to 659 MPa. For a strain rate of 0.008 s^−1^, the trend was similar, although the strength values in the range of 1000–1050 °C remained relatively high. These results indicate that the temperature of early deformation determines the trade-off between the material’s hardening and its ability to undergo further deformation.

Elongation behavior is also important in terms of the alloy’s expected response to intense thermal and mechanical stresses. Very low elongation obtained after deformation in the 700–800 °C range indicates the material’s limited ability to locally accommodate deformation, which may promote stress concentration and crack initiation during cyclic thermal or thermomechanical loading. In contrast, a marked increase in elongation above 900 °C—and particularly after deformation in the 1000–1200 °C range—indicates an improvement in plastic deformation capacity associated with dynamic recovery and recrystallization. Increased plasticity may limit the tendency for strain localization during thermomechanical processing and transient thermal loading (Figure 9). It should be emphasized, however, that the elongation results presented were obtained in a monotonic tensile test and should not be interpreted as a direct measure of resistance to thermal or thermomechanical fatigue. Assessing the durability of the alloy in aerospace and power generation applications would require conducting dedicated cyclic tests under conditions corresponding to actual temperatures, strain ranges, holding times, and operating environments.

### 3.6. Hardness

**Figure 10:**
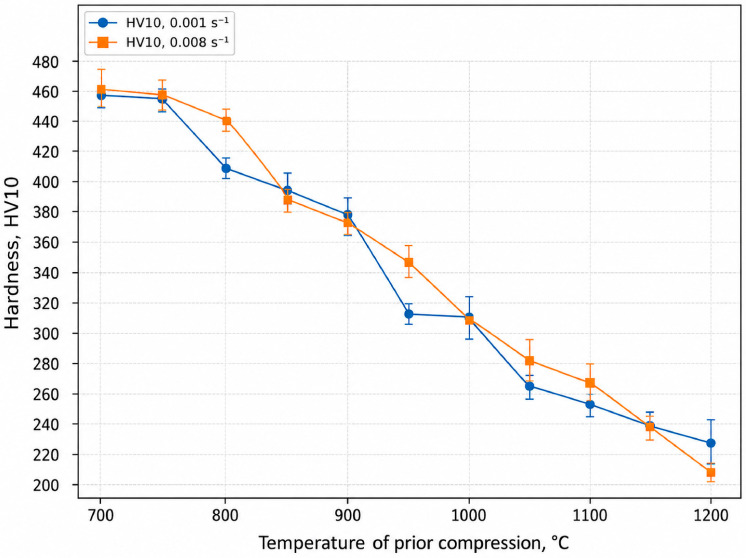
Changes in HV10 hardness of Haynes 282 alloy after deformation at different temperatures and strain rates. Horizontal reference lines indicate the hardness of the alloy in the as-cast condition and after solution treatment.

The hardness of the material after deformation was strongly dependent on the process temperature (Table 5, Figure 10). The highest values were obtained after compression at 700–800 °C, where the average hardness was 408–459 HV10 for 0.001 s^−1^ and 441–462 HV10 for 0.008 s^−1^. In this range, the microstructure remained heavily deformed, and the contribution of recovery and recrystallization processes was limited. As the temperature increased, the hardness decreased systematically, reaching 228 HV10 for 0.001 s^−1^ and 206 HV10 for 0.008 s^−1^ after compression at 1200 °C. The changes in hardness correlate well with the stress values determined from the compression tests and with the tensile test results. The high hardness at lower temperatures resulted from dislocation accumulation and the effect of precipitates on dislocation motion. The decrease in hardness at 900–1000 °C corresponded to intensified recovery and dynamic recrystallization, while the further decrease at 1050–1200 °C was associated with more complete recrystallization and grain growth. The relatively small decrease in hardness between 800 °C and 850 °C, from 408 to 395 HV10 at a strain rate of 0.001 s^−1^, indicates that the material still retained a strongly deformed microstructure, with only a limited contribution from recovery and the earliest signs of recrystallization. In contrast, the pronounced decrease between 900 °C and 950 °C, from 378 to 313 HV10, coincided with the onset and increasing contribution of dynamic recrystallization observed in the microstructural analysis. The formation of dynamically recrystallized regions reduced the stored deformation energy and the degree of dislocation accumulation, resulting in the marked hardness decrease.

### 3.7. Microstructural Evolution

The microstructural analysis performed in the present study was qualitative. A separate macrostructural comparison before and after dynamic recrystallization was not performed; therefore, the structural evolution was evaluated at the microstructural level using LM, SEM, and TEM observations. Therefore, no numerical values of dislocation density, recrystallized fraction, grain-growth rate, or grain-size ratio are reported. The interpretation of dynamic recovery, dynamic recrystallization, and grain growth is based on the observed microstructural features and their correlation with the corresponding flow behavior.

**Figure 11:**
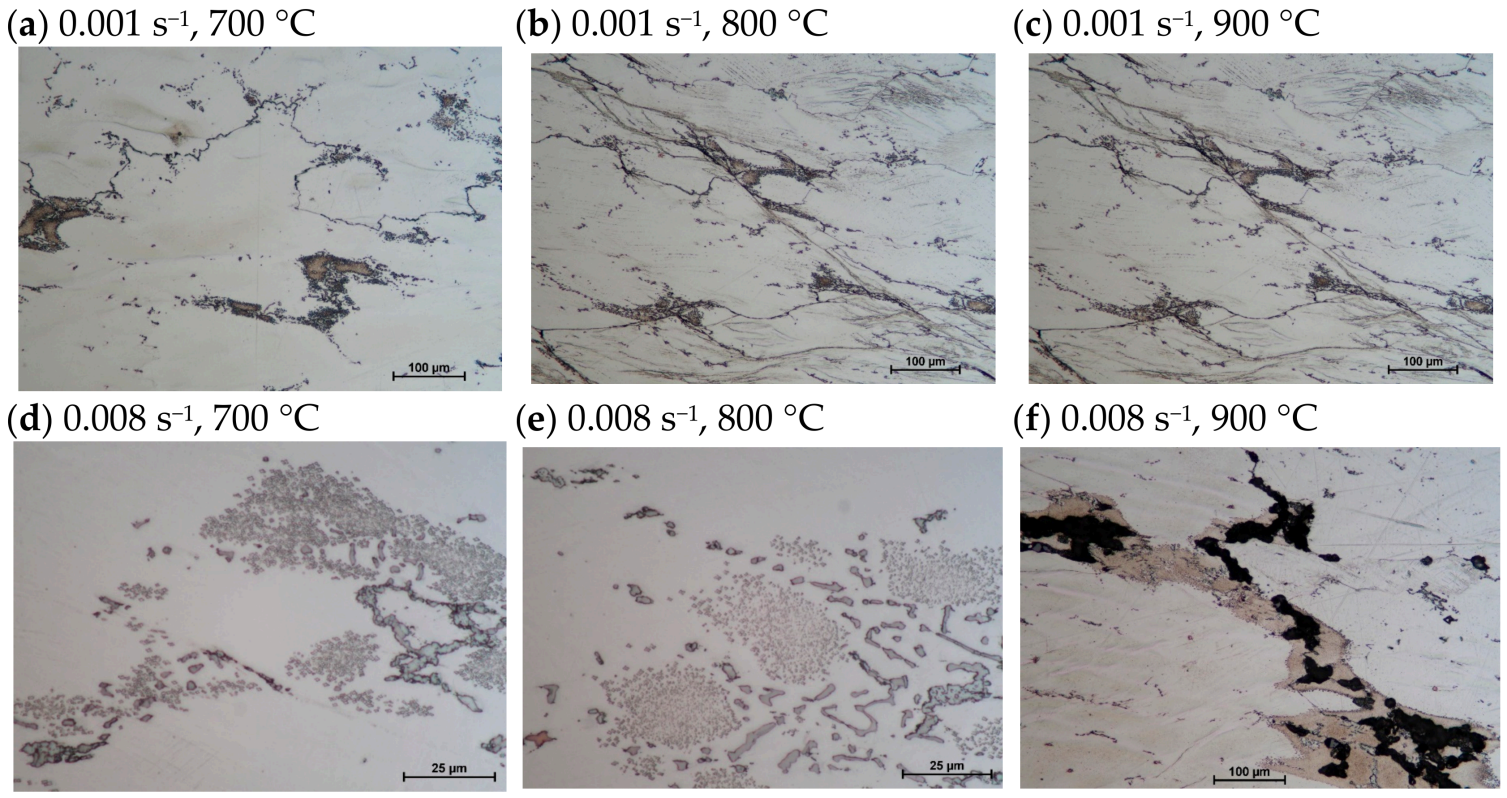
Representative LM images illustrating the microstructural evolution of the Haynes 282 superalloy after compression in the temperature range of 700–900 °C as a function of temperature and strain rate: (**a**–**c**) 0.001 s^−1^; (**d**–**f**) 0.008 s^−1^. The images show the transition from a severely deformed and locally discontinuous microstructure to a recrystallized and then coarse-grained microstructure.

LM, SEM, and TEM observations revealed a distinct sequence of microstructural changes as the deformation temperature increased. In the 700–800 °C range, a heavily deformed microstructure of primary grains dominated, featuring distinct slip bands, deformation localization, and regions with pronounced dislocation contrast (Figure 11). In specimens deformed at a higher strain rate of 0.008 s^−1^, local discontinuities and microcracks were additionally observed, indicating that the rate of defect accumulation exceeded the material’s ability to relax them (Figure 11e,f).

**Figure 12:**
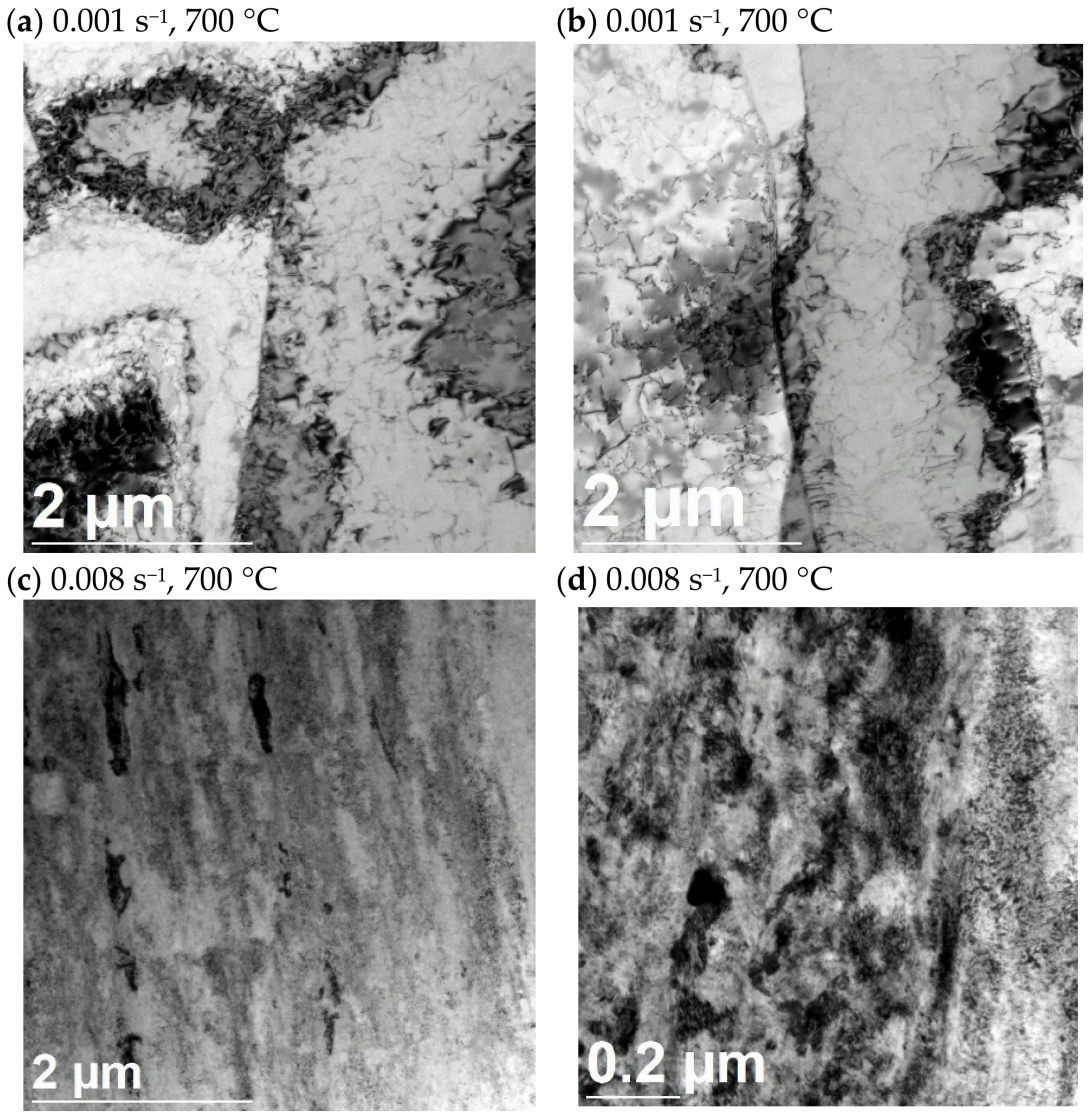
Representative BF-TEM micrographs of the Haynes 282 superalloy after hot compression at 700 °C: (**a**,**b**) strain rate of 0.001 s^−1^ and (**c**,**d**) strain rate of 0.008 s^−1^. The micrographs reveal a heavily deformed microstructure with pronounced dislocation structures, deformation bands, and localized strain. The higher strain rate results in more pronounced deformation localization and a greater accumulation of lattice defects owing to the limited contribution of thermally activated recovery processes.

To elucidate the deformation mechanisms operating at low temperatures, representative TEM observations of specimens compressed at 700 °C are presented in Figure 12. Regardless of the applied strain rate, the microstructure is characterized by pronounced dislocation structures and pronounced deformation localization. In specimens deformed at 0.001 s^−1^ (Figure 12a,b), dislocations are arranged into dense tangles and elongated deformation bands, indicating intensive strain hardening with only limited recovery. In contrast, deformation at the higher strain rate (0.008 s^−1^) (Figure 12c,d) results in more pronounced dislocation tangles and deformation bands and more pronounced deformation bands, reflecting the reduced time available for thermally activated recovery processes. No well-developed subgrain structure or dynamically recrystallized grains were observed under these conditions, indicating that strain hardening remained the dominant deformation mechanism at 700 °C.

**Figure 13:**
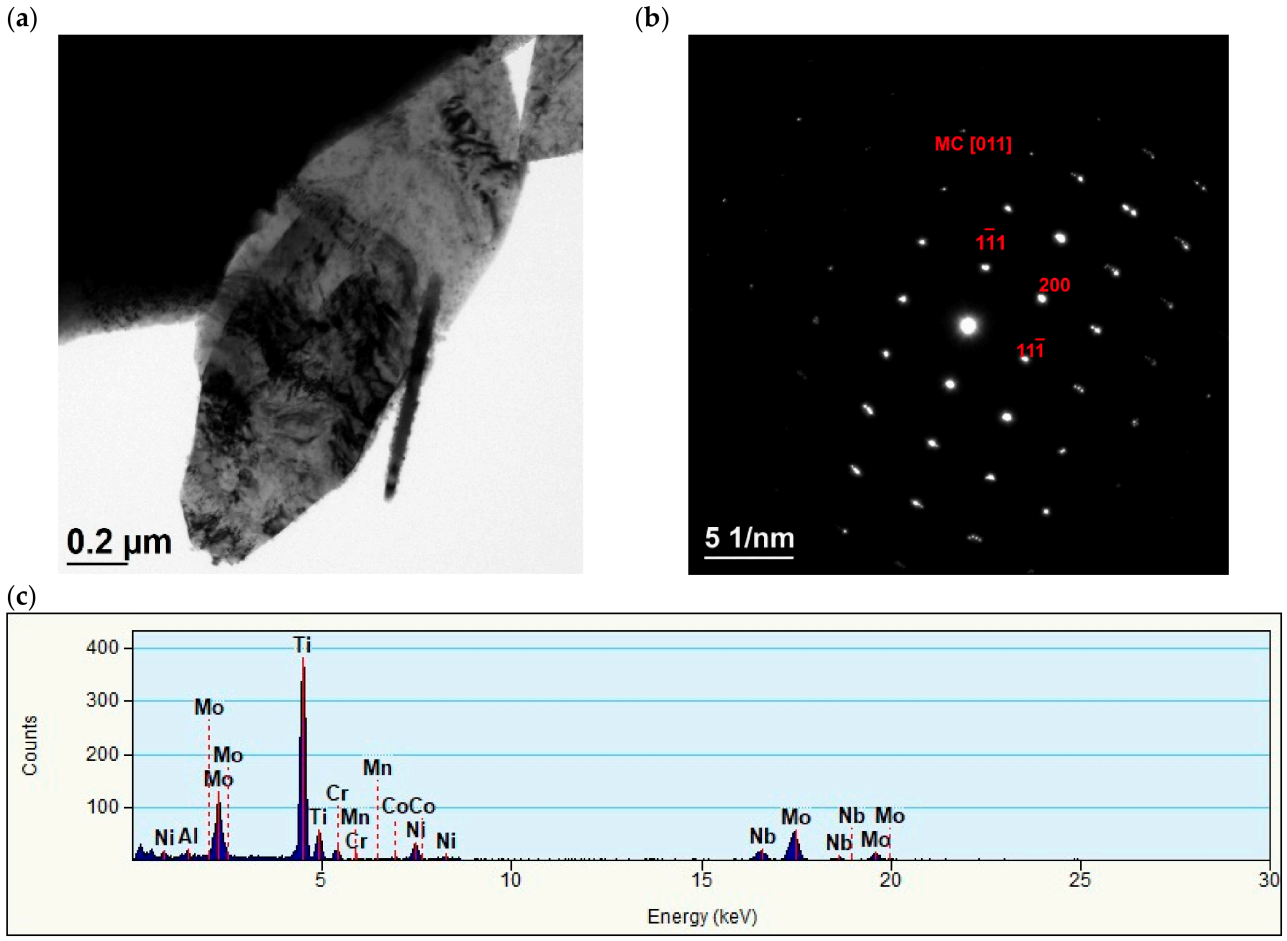
TEM characterization of a representative secondary particle observed in the Haynes 282 superalloy after compression at 700 °C and a strain rate of 0.008 s^−1^: (**a**) BF-TEM image of the particle embedded in the deformed matrix; (**b**) corresponding SAED pattern indexed as an MC-type carbide; (**c**) EDS spectrum confirming enrichment in Ti, Mo, and Nb.

To further characterize the secondary particles observed after deformation at 700 °C and a strain rate of 0.008 s^−1^, detailed TEM analysis was performed (Figure 13). The BF-TEM image (Figure 13a) reveals a coarse angular precipitate embedded within the heavily deformed matrix. The particle is surrounded by a high density of lattice defects, indicating a strong interaction between the precipitate and moving dislocations during plastic deformation.

The corresponding SAED pattern (Figure 13b) together with the EDS spectrum (Figure 13c) indicates that the particle is enriched primarily in Ti, with additional enrichment in Mo and Nb. The diffraction pattern is consistent with the crystallographic characteristics of an MC-type carbide, which is in agreement with the chemical composition obtained by EDS analysis. Such carbides are known to remain stable during hot deformation and contribute to strengthening by impeding dislocation motion and locally restricting grain boundary migration. The presence of these coarse primary carbides confirms that, although dynamic restoration processes were not yet well developed at 700 °C, the interaction between lattice defects and stable carbide particles played an important role in controlling the deformation behaviour of the alloy.

**Figure 14:**
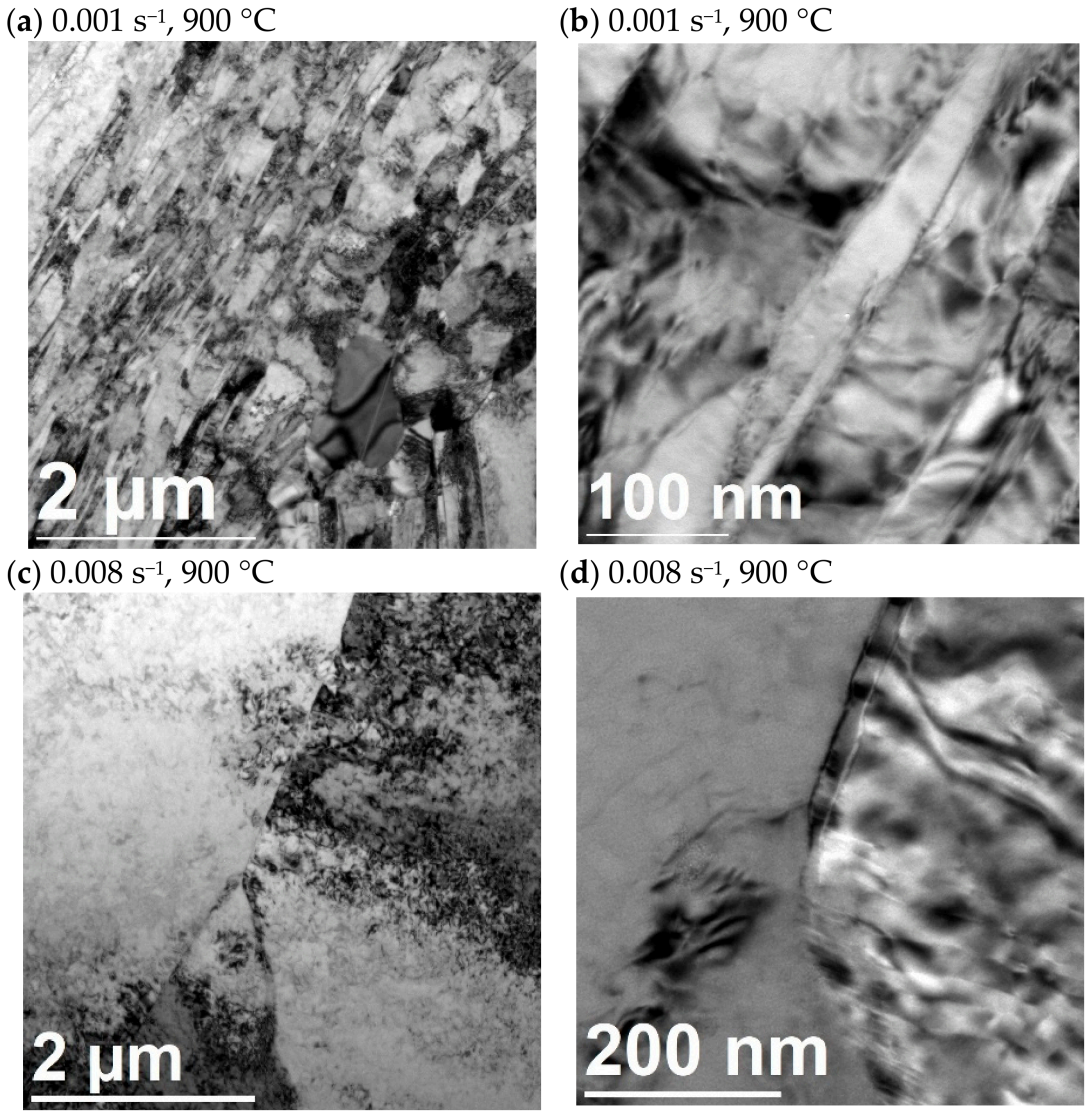
Representative BF-TEM micrographs illustrating the evolution of the deformation substructure of the Haynes 282 superalloy after compression at 900 °C: (**a**,**b**) strain rate of 0.001 s^−1^; (**c**,**d**) strain rate of 0.008 s^−1^. The images reveal the formation of dislocation cells, subgrain boundaries, and local regions with a reduced dislocation density, indicating the development of dynamic recovery and the initial stages of dynamic recrystallization. The higher strain rate results in a more pronounced dislocation structures and a less advanced restoration process.

At 900 °C, the first evidence of dynamic recrystallization became visible (Figure 14c,d). Fine equiaxed grains nucleated preferentially along the boundaries of the original grains and within deformation bands, was expected to be highest. Compared with lower deformation temperatures, the microstructure became more homogeneous, although regions containing elongated deformed grains were still present. These observations indicate that the alloy was in a transitional stage between strain hardening and the progressive development of dynamic recovery and dynamic recrystallization. TEM observations further confirmed the microstructural changes occurring at 900 °C (Figure 14). Compared with specimens deformed at 700 °C, the dislocation arrangement became more organized, with the formation of dislocation cells and well-defined subgrain boundaries. Local regions with less pronounced dislocation contrast were observed adjacent to heavily deformed areas, indicating the development of dynamic recovery and the initial stages of dynamic recrystallization. The coexistence of highly deformed regions and newly formed subgrains demonstrates that the alloy was in a transitional state between strain hardening and microstructural restoration. Similar features were observed at both strain rates; however, deformation at 0.008 s^−1^ resulted in a more pronounced dislocation structures and a less advanced recovery process owing to the shorter time available for thermally activated restoration mechanisms (Figure 14c,d).

**Figure 15:**
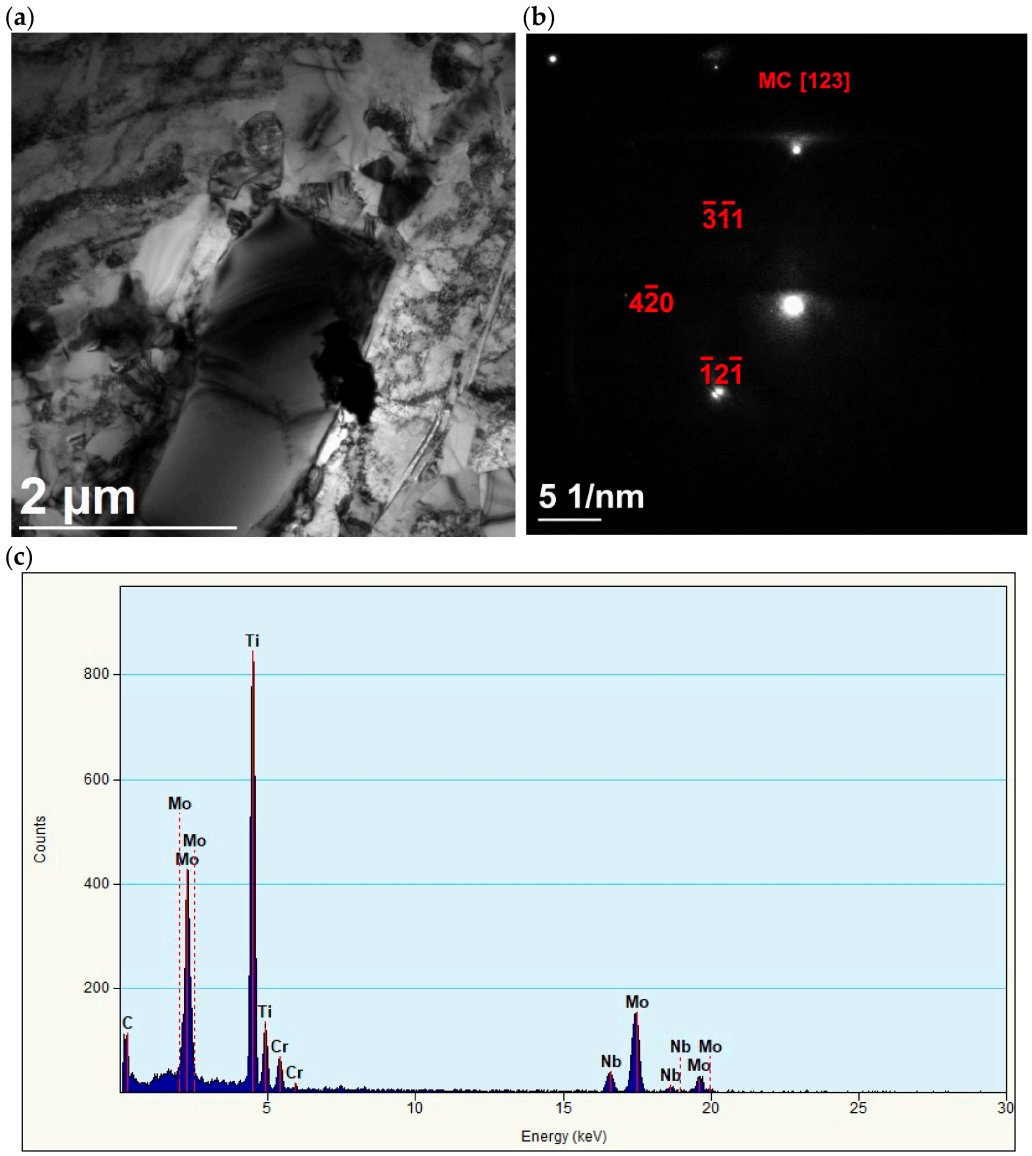
TEM characterization of a representative MC-type carbide observed in the Haynes 282 superalloy after compression at 900 °C and a strain rate of 0.001 s^−1^: (**a**) BF-TEM image showing a coarse carbide embedded in the partially recovered matrix; (**b**) indexed SAED pattern confirming the MC crystal structure; (**c**) EDS spectrum indicating enrichment in Ti, Mo, and Nb.

To further characterize the secondary particles present after deformation at 900 °C and a strain rate of 0.001 s^−1^, detailed TEM analysis was performed (Figure 15). The BF-TEM image (Figure 15a) reveals a coarse angular precipitate embedded within a partially recovered deformation substructure. Compared with the specimens deformed at 700 °C, the surrounding matrix exhibits a less pronounced dislocation contrast and a more organized arrangement of dislocations, consistent with the progressive development of dynamic recovery. The corresponding SAED pattern (Figure 15b) was indexed as an MC-type carbide, while the EDS spectrum (Figure 15c) confirmed that the particle was enriched predominantly in Ti, with additional contributions from Mo and Nb. These results indicate that the observed precipitate corresponds to a stable primary MC carbide, which remained undissolved during hot deformation at 900 °C.

The coarse carbide is surrounded by a pronounced dislocation accumulation, indicating a strong interaction between the precipitate and moving lattice defects. Such particles can act as effective obstacles to dislocation motion and locally retard grain boundary migration, thereby influencing the kinetics of dynamic recrystallization. Although dynamic recovery and the initial stages of dynamic recrystallization were already active at this temperature, the presence of stable MC carbides likely contributed to the heterogeneous distribution of stored deformation energy within the microstructure.

Although Haynes 282 may also contain secondary M_23_C_6_ carbides, particularly after aging, such particles were not conclusively identified in the present TEM analysis. Their presence cannot be excluded, but the available SAED and local EDS data were insufficient for unambiguous phase identification. This may result from their relatively low volume fraction, fine size, and preferential location along grain boundaries, which limited their representation in the investigated TEM foils. Although M_23_C_6_ carbides were not conclusively identified in the present study, their possible distribution along grain boundaries remains relevant to the high-temperature mechanical behavior of Haynes 282. A fine and discontinuous distribution of such carbides may restrict grain-boundary sliding and thereby contribute to improved creep resistance. In contrast, a continuous or semi-continuous grain-boundary distribution may locally reduce grain-boundary cohesion and increase susceptibility to intergranular cracking. Fine γ′ precipitates within the γ matrix are also expected to impede dislocation motion and limit time-dependent plastic deformation. The high deformation resistance and hardness observed at lower temperatures are qualitatively consistent with a strong contribution from γ′ strengthening. However, because the size, volume fraction, and spatial distribution of the γ′ precipitates and M_23_C_6_ carbides were not quantified, their influence on creep resistance and grain-boundary sliding cannot be directly assessed from the present results. Dedicated creep testing and quantitative microstructural characterization would be required to confirm these relationships.

**Figure 16:**
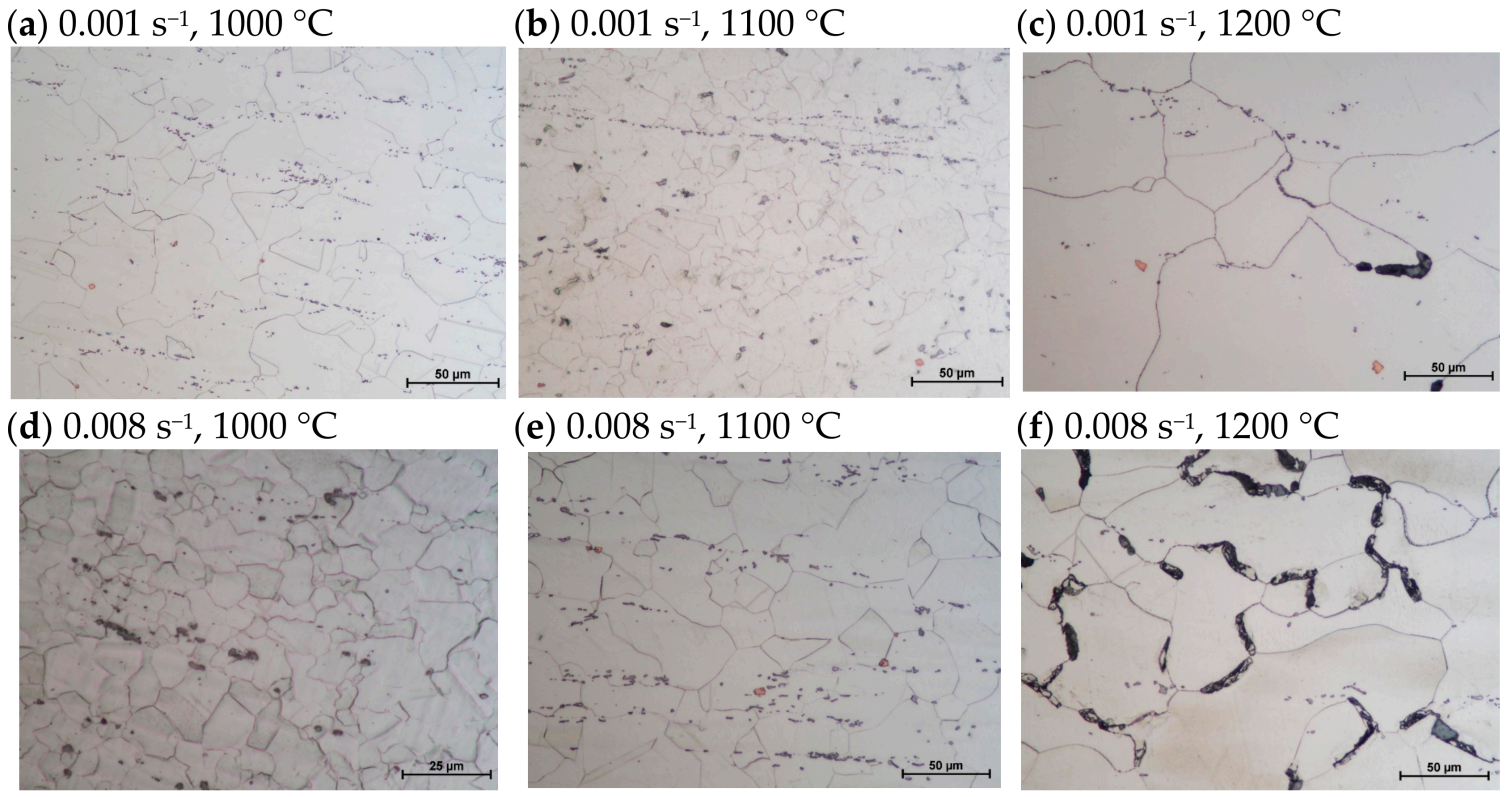
Representative LM images illustrating the microstructural evolution of the Haynes 282 superalloy after compression in the temperature range of 1000–1200 °C as a function of temperature and strain rate: (**a**–**c**) 0.001 s^−1^; (**d**–**f**) 0.008 s^−1^. The images show the transition from a severely deformed and locally discontinuous microstructure to a recrystallized and then coarse-grained microstructure.

**Figure 17:**
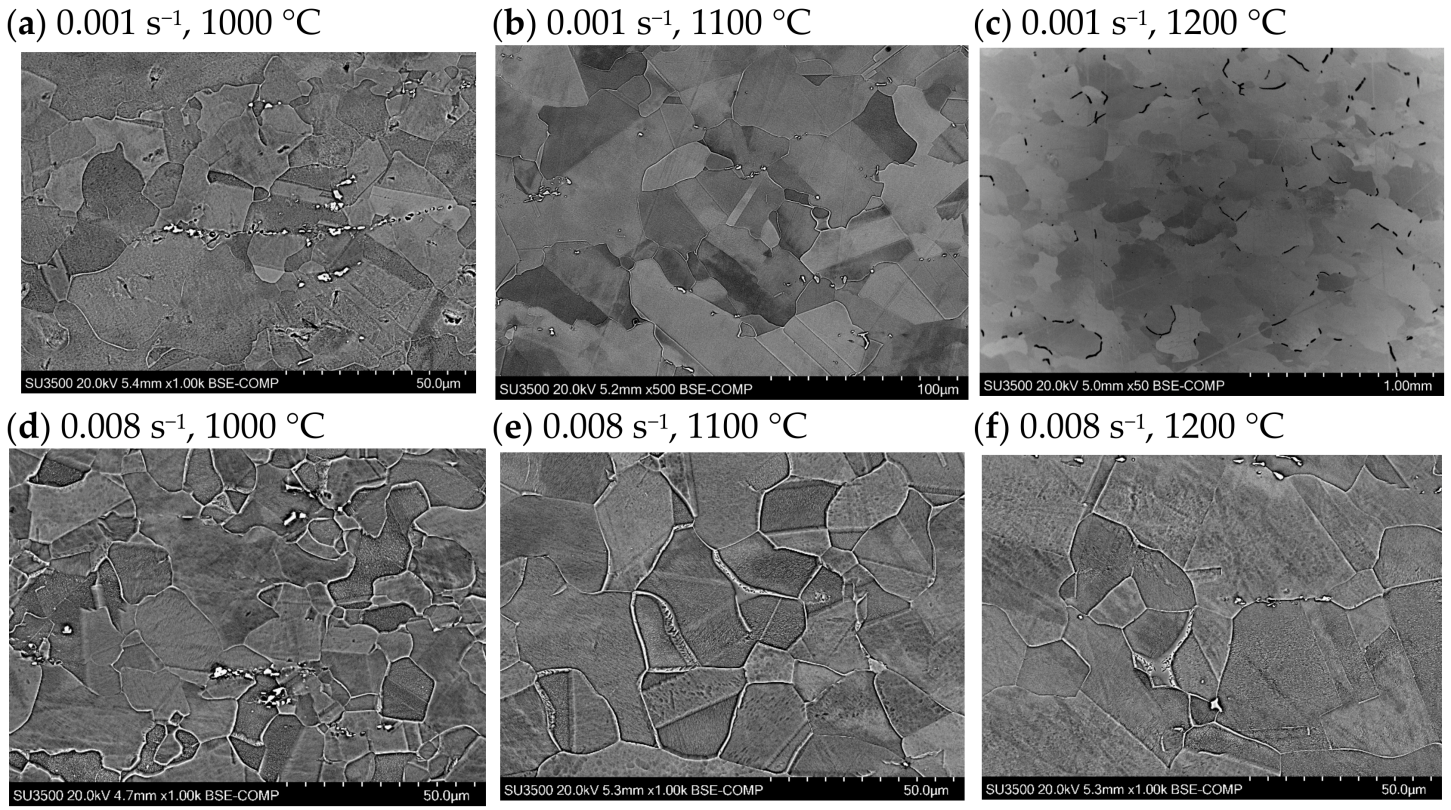
Representative SEM/BSE images of the Haynes 282 superalloy microstructure after compression: (**a**–**c**) strain rate of 0.001 s^−1^; (**d**–**f**) strain rate of 0.008 s^−1^. The comparison shows strain localization and the presence of secondary particles at lower temperatures, as well as the development and growth of recrystallized grains at higher temperatures.

At lower deformation temperatures, the microstructure retained strongly deformed, elongated, and locally distorted grains, together with pronounced deformation bands. At approximately 850–900 °C, the first fine equiaxed recrystallized regions became visible, mainly near grain boundaries and highly deformed areas. With increasing temperature to 1000–1050 °C, recrystallized grains were observed over increasingly extensive areas, and the microstructure became more homogeneous, while strongly deformed regions were less frequently observed. At the highest investigated temperatures, the increasing mobility of grain boundaries promoted grain coarsening. In the specimen deformed at 1200 °C and 0.008 s^−1^, dark, partly continuous features were observed along selected grain boundaries (Figure 16f). Based on LM observations alone, these features cannot be unambiguously identified as continuous or semi-continuous carbide films. If they correspond to grain-boundary carbides or other brittle secondary constituents, their continuity could locally reduce grain-boundary cohesion and promote intergranular crack initiation under tensile, creep, fatigue, or thermomechanical loading. Targeted SEM/EDS or TEM analysis would be required for definitive phase identification and assessment of their distribution. In the 1000–1200 °C range, recrystallized grains became increasingly evident (Figure 16). The microstructure became more homogeneous and fine-grained, while heavily deformed regions were observed less frequently. This temperature range can be considered the most technologically advantageous, as it allowed for a significant reduction in deformation resistance and limited the localization of deformation without excessive grain growth. Above 1000 °C, the recrystallization process already was observed over increasingly extensive areas, but a further increase in temperature led to increased grain boundary mobility and the development of a coarse-grained structure. At 1000–1050 °C, the stable primary MC carbides may have locally restricted grain-boundary migration and thereby contributed to limiting excessive grain coarsening. However, because their number density, spatial distribution, and pinning effect were not quantified, it cannot be concluded that they were sufficient to completely prevent abnormal grain growth. Supplementary SEM images are shown in Figure 17.

**Figure 18:**
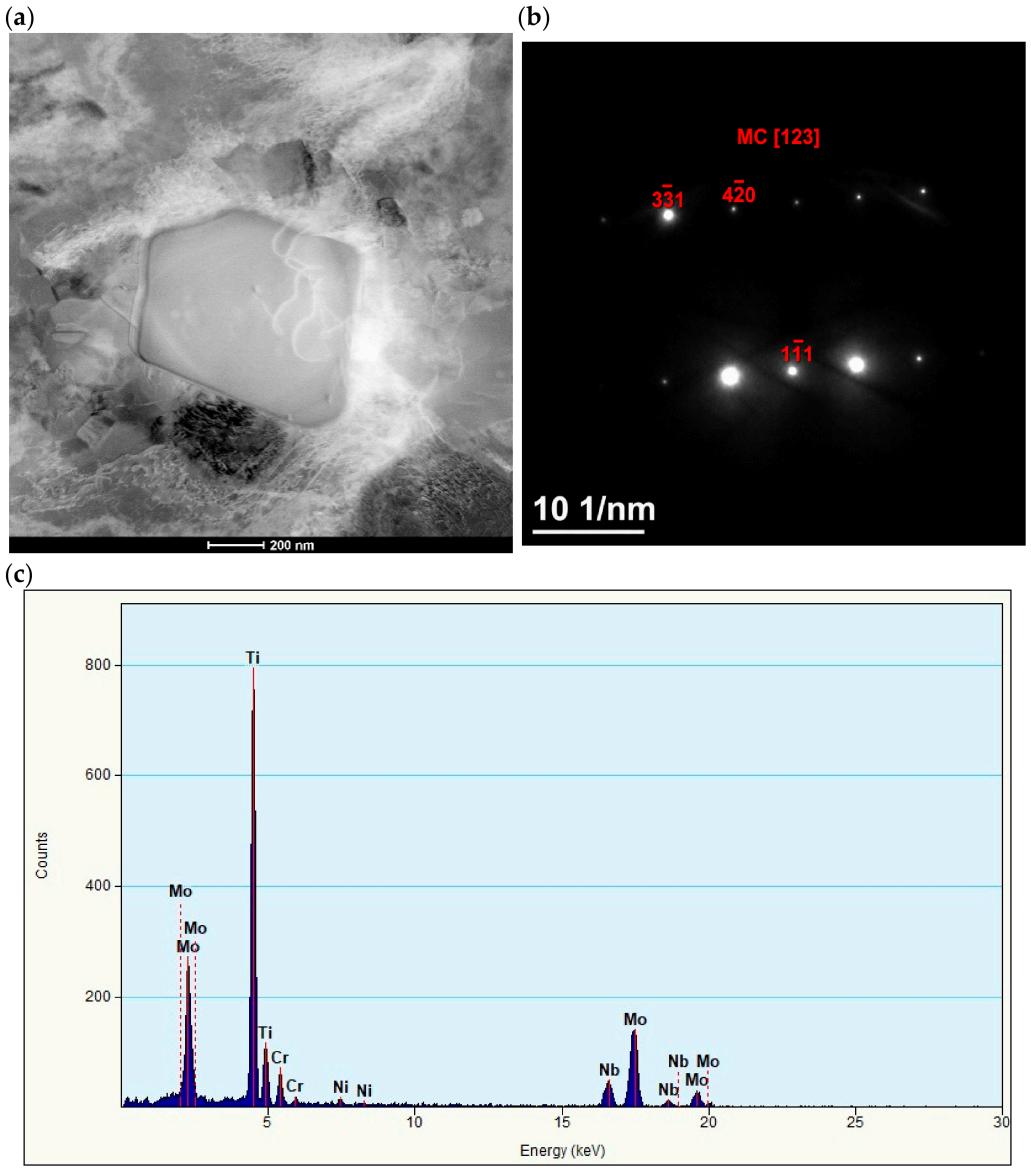
TEM characterization of a representative MC-type carbide observed in the Haynes 282 superalloy after compression at 1100 °C: (**a**) BF-TEM image showing a blocky carbide particle embedded in the recrystallized matrix; (**b**) corresponding SAED pattern indexed as an MC-type carbide; (**c**) EDS spectrum confirming enrichment mainly in Ti, with additional contributions from Mo and Nb.

At temperatures of 1100–1200 °C (Figure 17b–f), microstructural evolution was governed mainly by the growth of previously recrystallized grains. Individual secondary particles were still observed, predominantly along grain boundaries and within the recrystallized matrix. TEM/SAED/EDS analysis performed for the specimen deformed at 1100 °C confirmed the presence of a blocky MC-type carbide embedded in the recrystallized matrix (Figure 18). The corresponding SAED pattern was indexed as an MC carbide, while the EDS spectrum showed enrichment mainly in Ti, with additional contributions from Mo and Nb. No systematic fragmentation or redistribution of the primary MC carbides was demonstrated under the investigated thermomechanical-processing conditions. The observed particles retained a coarse and angular morphology and remained locally embedded in the deformed matrix. Therefore, the present results do not allow the effect of carbide fragmentation or redistribution on the mechanical properties to be evaluated. Such an assessment would require quantitative comparison of carbide size, number density, morphology, and spatial distribution before and after deformation. Although such particles may locally restrict dislocation motion and grain boundary migration, their ability to effectively inhibit grain growth is reduced at these high temperatures because of increased grain-boundary mobility. Consequently, this becomes increasingly important after recrystallization. TEM observations were therefore essential for linking the high-temperature microstructure with the deformation mechanisms, as they revealed dislocation rearrangement, subgrain formation, recrystallized regions, and the interaction between stable MC carbides and lattice defects.

## 4. Discussion

The results obtained indicate that cast Haynes 282 can be used as a starting material for further thermomechanical processing, provided that the quality of the casting is controlled and the heat treatment parameters are appropriately selected. The low porosity content in the analyzed bars confirms the possibility of obtaining material of sufficient quality for high-temperature testing. At the same time, the presence of even localized microporosity and secondary phases may significantly influence damage initiation during compression at lower temperatures, particularly at higher strain rates.

One of the most significant results of this study is the identification of the temperature range in which microstructural restoration mechanisms become active but do not yet lead to excessive grain growth. In the 700–800 °C range, the alloy exhibits very high flow and yield stresses. The high values of yield stress and hardness, together with the observed dislocation structures, indicate that the deformation behavior is controlled primarily by strain hardening and the interaction of dislocations with gamma prime precipitates and carbide phases. The calculated apparent activation energy additionally confirms the strong temperature dependence of the deformation process; however, it reflects the combined contribution of several deformation and restoration mechanisms over the investigated temperature range.

At temperatures of 850–1000 °C, a transition occurs to conditions in which dynamic recovery and dynamic recrystallization increasingly contribute to reducing the stored deformation energy. The formation of fine grains along the boundaries of the original grains and within deformation bands is consistent with preferential nucleation in regions of locally accumulated deformation. This is also the range in which a decrease in hardness and yield strength does not necessarily indicate material degradation, but rather a transition from a highly strengthened state to a state more favorable for further plastic forming. A temperature of 1000 °C appears to be particularly significant from a technological standpoint, as it corresponds to the pronounced development of dynamic recrystallization and the formation of a more homogeneous microstructure containing fine recrystallized regions. At this temperature, a significant increase in ductility was observed compared to specimens deformed at 700–900 °C, while maintaining acceptable strength values. A further increase in temperature to 1050–1200 °C improved the material’s deformability but simultaneously led to a decrease in hardness and strength. These changes were accompanied by increasing evidence of grain growth, particularly at the highest deformation temperatures.

A comparison of the two strain rates shows that the higher rate of 0.008 s^−1^ generally promotes higher flow resistance under selected lower-temperature conditions, while simultaneously limiting the time available for thermally activated restoration processes. As a result, at temperatures of 700–800 °C, the material is more susceptible to strain localization and microcrack initiation. At higher temperatures, the differences between the two strain rates generally become less pronounced, as the increased contribution of diffusion-assisted processes enables more effective dynamic recovery and recrystallization.

To place the present results in the context of previous investigations, Table 6 compares the principal findings of this study with selected literature data for Haynes 282. The comparison includes hardness, deformation or heat-treatment temperature, apparent activation energy, favorable processing conditions, and recrystallization temperature or dynamic recrystallization conditions. Direct comparison should be made cautiously because the available studies concern different initial material conditions, manufacturing routes, strain rates, heat treatments, and hardness-test loads. In addition, not all of the compared parameters were reported in every study.

The comparison presented in the table shows that the mechanical properties and recrystallization behavior of Haynes 282 alloy are strongly dependent on the initial condition of the material, its manufacturing process, the heat treatment applied, and its strain history. The hardness values reported in the literature for supersaturated, aged, cast, and welded material most often fall within the range of approximately 350–400 HV, whereas in this study, values exceeding 450 HV were obtained after deformation at 700–750 °C. Polkowska et al. [55] obtained a maximum hardness of 398 ± 4 HV after a two-stage aging process involving annealing at 950 °C for 2 h, followed by annealing at 780 °C for 8 h. The higher values obtained in this study can be attributed to the combined effect of precipitation hardening and the retained, highly deformed dislocation substructure. However, direct comparison requires caution due to differences in the material’s state, processing conditions, and the loads applied during hardness measurements.

The apparent activation energy values reported for the Haynes 282 alloy are also relatively high and show significant variation. Yao et al. [52] determined a value of 537.12 kJ/mol, while Shi et al. [24] reported a value of approximately 498 kJ/mol for GH4282, a nickel-based superalloy considered an equivalent of Haynes 282. In contrast, Semiatin et al. [57] obtained an apparent activation energy of 434 kJ/mol during studies of high-temperature plastic flow in Haynes 282. In the study by Semiatin et al. [57], this value was determined based on experiments conducted under continuous heating at a constant stress; therefore, it is not directly equivalent to the values obtained from classical isothermal compression tests. The authors also demonstrated that, after accounting for the temperature dependence of Young’s modulus, the corresponding activation energy for creep was approximately 386 kJ/mol.

The differences between the values of 434, 498, and 537.12 kJ/mol indicate that the activation energy should not be treated as a single, constant, and intrinsic material constant of the Haynes 282 alloy. Its value depends on the initial state of the material, the temperature range and strain rate, the test method used, the choice of characteristic stress, and the procedure for fitting the constitutive equation. In Haynes 282 alloy strengthened by γ′ phase precipitation, the apparent activation energy reflects the combined contribution of dislocation slip and climb, their interactions with γ′ precipitates and carbides, as well as dynamic recovery and dynamic recrystallization. Semiatin et al. [57] further indicated that the plastic flow of the studied alloy was limited by dislocation climb over γ′ precipitates.

Data on recrystallization indicate that its course also strongly depends on the material’s processing history. Eriksson et al. [24,54] demonstrated that during the compression of Haynes 282 at a temperature of approximately 1080 °C, dynamic recrystallization developed as strain increased. In their analysis of the relationship between peak stress and the Zener–Hollomon parameter, they used an activation energy value of Q = 498 kJ/mol derived from a previous study on Haynes 282.

A different behavior is observed in additively manufactured materials. Christofidou et al. [56] demonstrated that complete recrystallization of Haynes 282 alloy produced by laser powder melting required a temperature of approximately 1240 °C and a 2-h annealing period. Such a high temperature was associated with a different grain morphology and the presence of particles that restrict grain boundary movement. This demonstrates that the recrystallization temperature is not a single constant value specific to the alloy, but depends on the manufacturing method, the initial structure, the level of energy accumulated during prior processing, and the distribution of carbides and other particles.

Data from the manufacturer Haynes International [6] indicate that standard supersaturation of conventional Haynes 282 is performed in the range of 1121–1149 °C, followed by a two-stage aging process at 1010 °C for 2 h and 788 °C for 8 h. These data serve as an important reference point; however, they primarily apply to plastically worked material and cannot be directly equated with the optimal strain range for the cast material examined in this study.

Overall, the results of this study are consistent with the trend observed in the literature, according to which an increase in temperature promotes dynamic healing and recrystallization, while a further increase in temperature leads to increased grain boundary mobility and intensified grain growth. At the same time, the findings confirm that the favorable processing range cannot be considered universal for all forms and initial conditions of Haynes 282. The conditions indicated in this study apply primarily to the cast alloy under investigation that has undergone the heat treatment described.

From a practical standpoint, among the investigated conditions, the temperature range of approximately 900–1000 °C provided the most favorable balance between reduced deformation resistance, improved ductility, the development of dynamic recrystallization, and limited grain coarsening. This range promotes the development of dynamic recrystallization, a reduction in deformation resistance, and limited strain localization, a reduction in deformation resistance, and a limitation of strain localization, while avoiding the more pronounced grain coarsening observed at 1150–1200 °C. However, because the microstructural interpretation presented in this study is primarily qualitative, the proposed processing range should be regarded as an experimentally supported indication rather than a fully optimized processing window. The observed changes clearly indicate the possibility of controlling the microstructure of cast Haynes 282 by properly selected deformation temperature and strain rate. Future quantitative analyses of grain size, recrystallized fraction, and dislocation density could further strengthen this interpretation and provide a more precise description of the relationship between processing parameters and microstructural evolution.

## 5. Conclusions

1. Vacuum precision casting enabled the production of Haynes 282 superalloy bars with low porosity, and the vacuum furnace cooling method was selected as more suitable for further thermal-plastic studies.
2. DTA/DSC analysis revealed thermal effects in the range of 935.9–1379.8 °C, which allowed for the selection of heat treatment parameters and reduced the risk of overmelting of microstructural components during subsequent deformation.
3. Pre-deformation heat treatment at 990 °C for 1 h resulted in a decrease in hardness from approximately 330 HV10 in the as-cast condition to 259 HV10, which was consistent with partial dissolution of secondary precipitates and changes in the initial precipitation state of the γ matrix.
4. In the 700–800 °C range, the alloy exhibited the highest yield strength, high hardness, and strong strain hardening characteristics; at higher strain rates, increased strain localization and a risk of microcracking were observed.
5. Temperatures of 850–1000 °C favored the initiation and development of dynamic recrystallization. The most favorable compromise between reducing plastic resistance, maintaining a finer microstructure, and limiting the risk of cracking was achieved in the 900–1000 °C range.
6. At temperatures above 1050 °C, more extensive recrystallization was observed, accompanied by grain growth, decrease in hardness, and reduced tensile strength.
7. TEM characterization showed that the coarse primary MC carbides remained crystallographically and chemically stable under both investigated strain rates. The examined particles displayed comparable morphology, SAED patterns, and EDS spectra after deformation at 0.001 and 0.008 s^−1^, and no clear strain-rate-dependent carbide coarsening was observed. However, because carbide size and particle-size distribution were not quantified, this observation should be regarded as qualitative. The strain rate primarily affected the evolution of the surrounding dislocation substructure rather than producing an evident change in the primary MC carbides.
8. Compared with literature data for wrought Haynes 282, the investigated cast material exhibited higher apparent activation-energy values and elevated flow stresses, particularly in the low-temperature range. Dynamic recrystallization became increasingly evident at approximately 850–1000 °C, while deformation above 1050 °C promoted a further reduction in flow stress but also increased the tendency toward grain growth. From an industrial perspective, these results indicate that hot forming below approximately 900 °C may require high forming loads and may increase the risk of strain localization and cracking. Among the investigated conditions, the range of 900–1000 °C provided the most favorable balance between reduced deformation resistance, increasing recrystallization activity, and limited grain coarsening.
9. The results confirm the possibility of controlling the microstructure of cast Haynes 282 through a controlled sequence of casting, heat treatment, and thermomechanical processing. However, additional quantitative analyses of grain size, recrystallized fraction, and precipitate distribution, together with hot-deformation tests at strain rates of approximately 0.01–1 s−1, would further support the development and optimization of this processing route under conditions more representative of industrial forming.

## Figures and Tables

**Table 1:** Nominal chemical composition of the Haynes 282 superalloy used in the study.

Element	Ni	Cr	Co	Mo	Ti	Al	Fe	Mn	Si	C	B
Content, % by mass	balance	20.0	10.0	8.5	2.1	1.5	1.5	0.3	0.15	0.06	0.005

**Table 2:** Porosity fraction in rods cast from Haynes 282 superalloy depending on the cooling method. The results are presented as mean ± standard deviation calculated from seven randomly selected observation fields for each variant (*n* = 7).

Rod Diameter, mm	Porosity After Cooling in a Vacuum Furnace, %	Porosity After Air Cooling, %
10	0.036 ± 0.001	0.078 ± 0.004
12	0.160 ± 0.015	0.062 ± 0.006
16	0.088 ± 0.003	0.100 ± 0.034

**Table 3:** Effect of deformation temperature on the maximum stress and yield stress of the Haynes 282 alloy.

Deformation Temperature, °C	Strain Rate: 0.001 s^−1^		Strain Rate: 0.008 s^−1^	
	σ_max_	σ_0.2_	σ_max_	σ_0.2_
700	930	910	1100	1050
750	900	800	950	930
800	830	710	850	840
850	570	560	730	700
900	450	430	570	540
950	250	230	400	340
1000	130	120	190	175
1050	95	90	145	135
1100	80	70	115	105
1150	65	55	85	75
1200	50	40	62	60

**Table 4:** Mechanical properties of Haynes 282 alloy in the as-cast condition and after prior high-temperature compression. The results are presented as mean ± standard deviation based on three tensile microspecimens for each condition (*n* = 3).

Condition/Strain Rate ε, s^−1^	Deformation T, °C	R_p0.2_, MPa	R_m_, MPa	A, %
Condition after casting	-	556 ± 9.5	822 ± 8.7	20.0 ± 1.5
0.001	700	1106 ± 5.1	1153 ± 7.0	9.6 ± 0.8
0.001	800	1082 ± 13.1	1225 ± 4.4	2.4 ± 0.4
0.001	900	867 ± 6.6	1034 ± 11.1	11.5 ± 0.9
0.001	1000	452 ± 6.4	757 ± 5.3	29.6 ± 1.4
0.001	1050	538 ± 10.4	891 ± 10.3	38.0 ± 2.1
0.001	1200	382 ± 8.9	659 ± 9.8	47.6 ± 3.1
0.008	700	-	1274 ± 18.7	-
0.008	800	993 ± 4.4	1069 ± 11.9	3.9 ± 0.7
0.008	900	836 ± 6.1	1006 ± 9.7	12.3 ± 1.4
0.008	1000	648 ± 2.6	962 ± 8.7	21.5 ± 2.4
0.008	1050	595 ± 19.9	936 ± 11.2	32.8 ± 3.4
0.008	1200	334 ± 4.9	544 ± 5.9	38.6 ± 3.9

**Table 5:** Average HV10 hardness of specimens after deformation as a function of temperature and deformation rate.

T, °C	HV10 Strain Rate: 0.001 s^−1^	HV10 Strain Rate: 0.008 s^−1^
700	459.2 ± 4.7	462.2 ± 11.2
750	456.3 ± 2.4	458.7 ± 8.7
800	407.8. ± 4.4	441.0 ± 4.6
850	394.6 ± 3.9	389.2 ± 5.5
900	377.7 ± 8.1	372.7 ± 4.0
950	313.1 ± 4.7	345.9 ± 7.2
1000	311.4 ± 9.2	307.1 ± 6.8
1050	265.7 ± 7.1	280.3 ± 9.7
1100	253.2 ± 8.9	266.5 ± 8.4
1150	238.9 ± 3.4	237.7 ± 5.6
1200	227.8 ± 11.2	205.6 ± 3.7

**Table 6:** Comparison of the present findings with selected literature results for Haynes 282 and related γ′-strengthened nickel-based superalloys.

Study and Material Condition	Hardness	Investigated Deformation or Treatment Temperature	Activation Energy, Q	Recommended or Favorable Processing Temperature	Recrystallization Temperature or DRX Condition
Present study—investment-cast and heat-treated Haynes 282	459.2 ± 4.7 to 227.8 ± 11.2 HV10 at 0.001 s^−1^; 462.2 ± 11.2 to 205.6 ± 3.7 HV10 at 0.008 s^−1^	Hot compression at 700–1200 °C; 0.001 and 0.008 s^−1^	Temperature-dependent apparent Q; highest values of approximately 635–747 kJ mol^−1^ at 0.001 s^−1^ and 691–812 kJ mol^−1^ at 0.008 s^−1^ in the 700–800 °C range	Approximately 900–1000 °C	First microstructural evidence of DRX at approximately 900 °C; increasingly extensive recrystallization above 1000 °C; grain coarsening at 1150–1200 °C
Yao et al.—wrought Haynes 282 [52]	Not reported	Isothermal hot compression in sub-solvus and super-solvus ranges	537.12 kJ mol^−1^	High-temperature and low-strain-rate conditions favored homogeneous fine DRX; exact optimum range should be copied from the original processing map	DRX promoted by increasing temperature and decreasing strain rate; discontinuous DRX identified as the principal nucleation mechanism
Eriksson et al.—wrought Haynes 282 [50,54]	Not reported	Hot compression mainly at 1060, 1080, and 1120 °C; conditions below and above the secondary-carbide solvus were considered	Not reported	No single optimum processing window proposed; temperature and strain rate had a greater effect on recrystallization than grain-boundary carbides	At 1080 °C and 0.05 s^−1^, small DRX grains were observed at strain ε ≈ 0.1, before peak strain ε_p_ ≈ 0.15; full recrystallization was obtained at ε ≈ 1.5
Polkowska et al.—wrought and aged Haynes 282 [55]	Maximum of 398 ± 4 HV after 950 °C/2 h + 780 °C/8 h	Multi-variant two-stage aging: 900–1100 °C followed by 680–880 °C	Not reported	950 °C/2 h + 780 °C/8 h gave the highest hardness among the investigated variants; this is an aging condition, not a hot-working window	Not reported
Christofidou et al.—LPBF Haynes 282 [56]	Not reported in the form directly comparable with HV10 after hot compression	Post-build heat treatments including 1150–1250 °C solution treatments	Not reported	A suitable heat treatment enabled properties comparable with conventionally produced material	Minimum temperature of approximately 1240 °C for 2 h was required to fully recrystallize the LPBF material
Haynes International—conventional wrought Haynes 282 [6]	Not reported as a single comparable value	Typical solution annealing at 1121–1149 °C; subsequent aging at 1010 °C and 788–802 °C	Not reported	1121–1149 °C is recommended for solution annealing rather than for deformation	

## Data Availability

The original contributions presented in this study are included in the article. Further inquiries can be directed to the corresponding author.
